# In-depth virological and immunological characterization of HIV-1 cure after CCR5Δ32/Δ32 allogeneic hematopoietic stem cell transplantation

**DOI:** 10.1038/s41591-023-02213-x

**Published:** 2023-02-20

**Authors:** Björn-Erik Ole Jensen, Elena Knops, Leon Cords, Nadine Lübke, Maria Salgado, Kathleen Busman-Sahay, Jacob D. Estes, Laura E. P. Huyveneers, Federico Perdomo-Celis, Melanie Wittner, Cristina Gálvez, Christiane Mummert, Caroline Passaes, Johanna M. Eberhard, Carsten Münk, Ilona Hauber, Joachim Hauber, Eva Heger, Jozefien De Clercq, Linos Vandekerckhove, Silke Bergmann, Gábor A. Dunay, Florian Klein, Dieter Häussinger, Johannes C. Fischer, Kathrin Nachtkamp, Joerg Timm, Rolf Kaiser, Thomas Harrer, Tom Luedde, Monique Nijhuis, Asier Sáez-Cirión, Julian Schulze zur Wiesch, Annemarie M. J. Wensing, Javier Martinez-Picado, Guido Kobbe

**Affiliations:** 1 Department of Gastroenterology, Hepatology and Infectious Diseases, Düsseldorf University Hospital, Medical Faculty, Heinrich Heine University, Düsseldorf, Germany; 2 Institute of Virology, University and University Hospital Cologne, University of Cologne, Cologne, Germany; 3 German Center for Infection Research, Partner Site Bonn-Cologne, Cologne, Germany; 4 Infectious Diseases Unit, I. Department of Medicine, University Medical Center Hamburg-Eppendorf, Hamburg, Germany; 5 Institute of Virology, Düsseldorf University Hospital, Medical Faculty, Heinrich Heine University, Düsseldorf, Germany; 6 IrsiCaixa AIDS Research Institute, Barcelona, Spain; 7 Center for Biomedical Research in Infectious Diseases (CIBERINFEC), Carlos III Health Institute, Madrid, Spain; 8 Vaccine and Gene Therapy Institute and Oregon National Primate Research Center, Oregon Health and Science University, Beaverton, OR USA; 9 Translational Virology, Department of Medical Microbiology, University Medical Center Utrecht, Utrecht, the Netherlands; 10 Institut Pasteur, Paris Cité University, HIV Inflammation and Persistence, Paris, France; 11 German Center for Infection Research, Partner Site Hamburg-Lübeck-Borstel-Riems, Hamburg, Germany; 12 Infectious Diseases and Immunodeficiency Section, Department of Internal Medicine 3, Universitätsklinikum Erlangen, Friedrich-Alexander Universität Erlangen-Nürnberg, Erlangen, Germany; 13 Leibniz Institute of Virology, Hamburg, Germany; 14 HIV Cure Research Center and Department of General Internal Medicine and Infectious Diseases, Ghent University Hospital, Ghent, Belgium; 15 Institute for Transplant Diagnostics and Cell Therapeutics, Düsseldorf University Hospital, Medical Faculty, Heinrich Heine University, Düsseldorf, Germany; 16 Department of Hematology, Oncology and Clinical Immunology, Medical Faculty, Düsseldorf University Hospital, Heinrich Heine University, Düsseldorf, Germany; 17 Ezintsha, University of the Witwatersrand, Johannesburg, South Africa; 18 University of Vic-Central University of Catalonia, Barcelona, Spain; 19 Catalan Institution for Research and Advanced Studies, Barcelona, Spain; 20 Present Address: Bavarian Nordic, Martinsried, Germany; 21 Present Address: Helmholtz Center for Infection Research, Helmholtz Institute for One Health, Greifswald, Germany; 22 Present Address: University Children’s Research, UCR@Kinder-UKE, University Medical Center Hamburg-Eppendorf, Hamburg, Germany; 23 Germans Trias i Pujol Research Institute, Barcelona, Spain

**Keywords:** HIV infections, Translational immunology, Bone marrow transplantation, Viral reservoirs

## Abstract

Despite scientific evidence originating from two patients published to date that CCR5Δ32/Δ32 hematopoietic stem cell transplantation (HSCT) can cure human immunodeficiency virus type 1 (HIV-1), the knowledge of immunological and virological correlates of cure is limited. Here we characterize a case of long-term HIV-1 remission of a 53-year-old male who was carefully monitored for more than 9 years after allogeneic CCR5Δ32/Δ32 HSCT performed for acute myeloid leukemia. Despite sporadic traces of HIV-1 DNA detected by droplet digital PCR and in situ hybridization assays in peripheral T cell subsets and tissue-derived samples, repeated ex vivo quantitative and in vivo outgrowth assays in humanized mice did not reveal replication-competent virus. Low levels of immune activation and waning HIV-1-specific humoral and cellular immune responses indicated a lack of ongoing antigen production. Four years after analytical treatment interruption, the absence of a viral rebound and the lack of immunological correlates of HIV-1 antigen persistence are strong evidence for HIV-1 cure after CCR5Δ32/Δ32 HSCT.

## Abstract (web summary)

The recipient of an allogeneic stem cell transplant from a CCR5Δ32/Δ32 donor shows evidence of HIV type 1 cure, including the absence of a viral rebound over 4 years after stopping antiretroviral treatment.

## Main

Human immunodeficiency virus type 1 (HIV-1) persists in the body during antiretroviral therapy (ART) in latently infected CD4^+^ T cells, but allogeneic hematopoietic stem cell transplantation (HSCT) has been shown to substantially reduce the viral reservoir^1,2^. However, some of the reservoir-harboring immune cells are extremely long-lived^3^, partially resistant to chemotherapy regimens used during HSCT procedures and can cause viral rebound on analytical treatment interruption (ATI)^4,5^. Notably, both cases of successful HIV-1 cure published so far—the ‘London patient’ (IciStem no. 36) and the ‘Berlin patient’—received a CCR5Δ32/Δ32 allograft^6,7^ conferring extended resistance to HIV-1 due to the absence of surface expression of the CCR5 coreceptor.

**Fig. 1:**
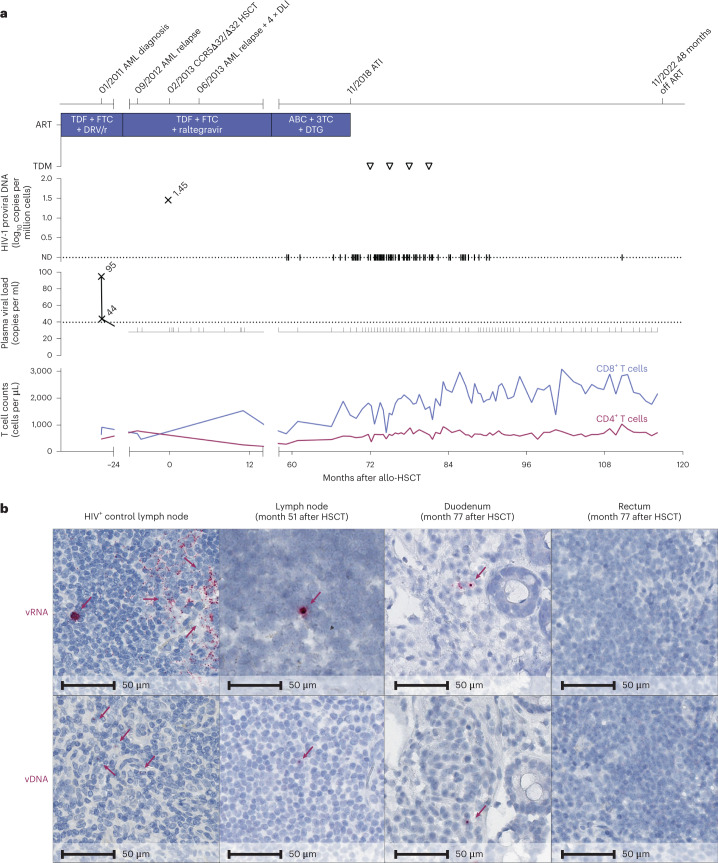
Clinical course and HIV-1 reservoir before and after ATI. **a**, Clinical events, ART regimen, HIV-1 plasma viral load, proviral load from PBMC and CD4^+^ and CD8^+^ counts. The triangles indicate negative results in the therapeutic drug monitoring (TDM) 3, 6, 9 and 12 months after ATI. ABC, abacavir; ND, not detectable. **b**, In situ hybridization assays for viral DNA (vDNA) and viral RNA (vRNA) revealed HIV-1 DNA and RNA traces in lymph node tissue (month 51) and duodenum but not rectum biopsies (month 77). Left panels: untreated HIV^+^ control patient.

In this study, we provide a detailed longitudinal virological and in-depth immunological analysis of the peripheral blood and tissue compartments of a 53-year-old male patient (IciStem no. 19)^8^, alive and in good health 117 months after CCR5Δ32/Δ32 allogeneic HSCT and 48 months after ATI. The patient was diagnosed to be HIV-1 clade B positive in January 2008 and presented with a CD4^+^ T cell count of 964 cells per μl and an HIV-1 plasma viral load of 12,850 copies per ml (Centers for Disease Control and Prevention A1, which was no indication for initiation of ART according to the national guidelines at that time). In October 2010, an ART regimen with tenofovir disoproxil fumarate (TDF), emtricitabine (FTC) and darunavir and ritonavir (DRV/r) was initiated (503 CD4^+^ T cells per μl and 35,303 HIV-1 copies per ml), resulting in a continuously suppressed plasma viral load (Fig. 1a). In January 2011, the patient was diagnosed with acute myeloid leukemia (AML) M2 according to the French–American–British classification, which carried an inversion of chromosome 16 at p13q22, resulting in the CBFB–MYH11 fusion protein. The patient achieved hematological complete remission after chemotherapy, which included idarubicin, cytarabine, etoposide induction therapy and three high-dose cytarabine (HiDAC) consolidation cycles. To avoid drug–drug interactions, DRV/r was switched to raltegravir in March 2011.

**Extended Data Fig. 1:**
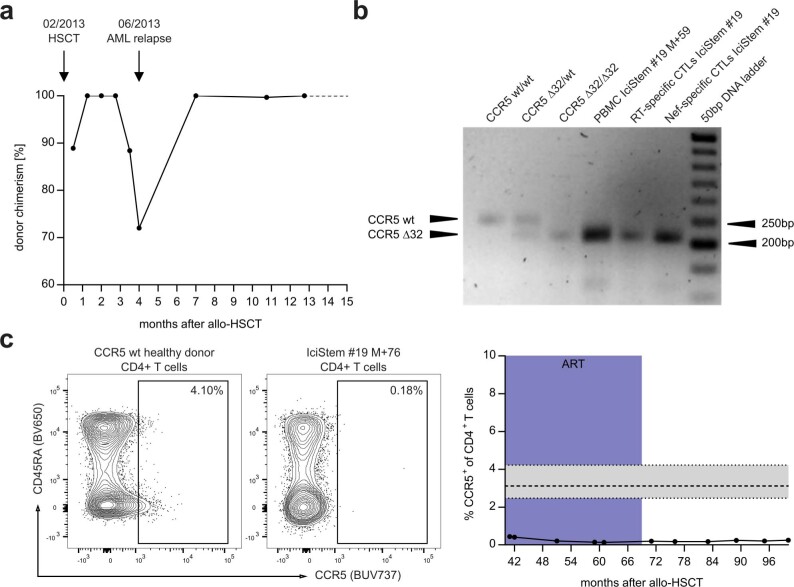
Donor chimerism and CCR5 genotype and phenotype in PBMC after HSCT. **a**, The donor chimerism after HSCT was genotypically assessed from a set of 13 genes. Full (100%) donor chimerism was first achieved 34 days after HSCT then dropped to 72% during the second relapse of AML but returned to 100% after successful therapy with 5-azacitidine and donor lymphocytes, and was sustained afterwards. HSCT, hematopoietic stem cell transplantation; AML, acute myeloid leukemia. **b**, In CCR5 genotyping with agarose gel electrophoresis of CCR5-PCR products, PBMC of the patient from month 35 after HSCT as well as *in vitro* generated HIV-1-specific CTL lines showed the homozygous CCR5Δ32 mutation. PBMC and B-cell lines from CCR5wt/wt, CCR5Δ32/wt, and CCR5Δ32/Δ32 individuals and a 50 bp DNA ladder served as controls. PBMC, peripheral blood mononuclear cells; wt, wildtype; RT, reverse transcriptase. **c**, CCR5 expression was lost from peripheral blood CD4+ T cells after HSCT in flow cytometric analyses. See also data from months 41–59 after HSCT for IciStem no. 19 as published in^8^. A reference range from a healthy cohort (n = 8) is given in grey as median with IQR. ART, antiretroviral therapy. Source data

**Extended Data Fig. 2:**
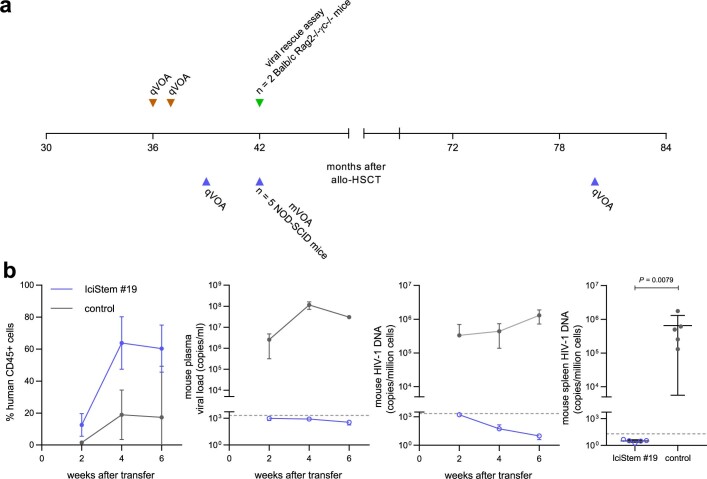
Timeline of viral outgrowth assays. **a**, Timeline of the viral outgrowth assays. Orange arrows indicate assays performed at the Heinrich Heine University Duesseldorf, green arrows indicate assays performed at the Leibniz Institute of Virology, and blue arrows assays at the IrsiCaixa AIDS Research Institute. All qVOA showed negative results and an *in vivo* viral rescue assay in Balb/c Rag2^−/−^γc^−/−^ mice showed negative results in the immunohistochemical stainings for HIV-1 p24 Ag in the spleens, livers, and lymph nodes (Supplementary Fig. 1). HSCT, hematopoietic stem cell transplantation; ATI, analytical treatment interruption; qVOA, quantitative viral outgrowth assay; mVOA, murine viral outgrowth assay. **b**, A mVOA in n = 5 biologically independent NOD-scid mice from month 42 after HSCT showed good engraftment of the transferred CD4+ T cells and undetectable plasma HIV-1 RNA and cellular HIV-1 DNA in the blood of the mice. Filled symbols indicate detectable values and open symbols indicate the limit of detection for undetectable values. After six weeks, HIV-1 DNA in the spleen of the mice was undetectable when infused with the IciStem no. 19 patient cells (blue) compared to an HIV-1 positive control patient under ART (grey) (two-tailed Mann-Whitney test; *P* = 0.0079). Data are shown as mean with standard deviation.

In September 2012, the patient experienced an AML relapse but achieved a second complete remission after treatment with the A-HAM chemotherapy regimen (retinoic acid, HiDAC, mitoxantrone) and a second cycle of HiDAC. A systematic search identified a 10/10 HLA-matched (no mismatches in HLA-A, HLA-B, HLA-C, HLA-DR and HLA-DQ loci) unrelated female stem cell donor with a homozygous CCR5Δ32 mutation (Extended Data Table 1). After reduced-intensity conditioning with fludarabine, treosulfan and anti-thymocyte globulin, 8.74 × 10^6^ unmodified CD34^+^ peripheral blood stem cells per kg of body weight were transplanted in February 2013. Immunosuppressive therapy consisted of cyclosporine and mycophenolate mofetil and was later changed to tacrolimus monotherapy. In June 2013, the patient experienced a second AML relapse. He achieved molecular oncological remission a third time after eight cycles with 5-azacytidine and four donor lymphocyte infusions (DLIs; 1 × 10^6^, 10 × 10^6^ and twice 50 × 10^6^ donor T cells per kg of body weight). Thirty-four days after HSCT, full donor chimerism was established and retained except for a short period during the second relapse at months 3 and 4 after HSCT (Extended Data Fig. 1a). In 2014, elevated liver enzymes caused discussion about possible hepatic graft versus host disease (GvHD), but a liver biopsy in May 2015 was interpreted as drug-induced liver injury. In July 2014, the patient also experienced reactivation of cytomegalovirus (CMV) (duodenal ulcer) and herpes simplex virus 2 (genital ulcers and cerebral vasculitis) and human herpesvirus 8 and Epstein–Barr virus (viremia) but recovered after specific antiviral treatment of the CMV and herpes simplex virus 2 infections. After DLI, mild chronic GvHD of the eyes with bilateral keratoconjunctivitis sicca developed that persists until today. ART was continued throughout and proviral HIV-1 DNA and HIV-1 RNA remained undetectable despite intensified testing in clinical routine assays (Fig. 1a). However, multiple assessments of the HIV-1 viral reservoir in the peripheral blood and lymphoid and gut tissue before and after ATI revealed sporadic HIV-1 DNA traces at several time points, with a higher frequency compared to HIV-1-negative donors and no-template controls (Extended Data Table 2). Although rare, residual HIV-1 DNA and HIV-1 RNA were also detected by in situ hybridization (DNAscope and RNAscope assays) from histological sections of inguinal lymph node tissue from month 51 and some gut biopsies from month 77 (Fig. 1b); the number of HIV-1 RNA^+^ cells (2.61 ± 0.13 HIV-1 RNA^+^ cells per 10^5^ cells) and HIV-1 DNA^+^ cells (5.08 ± 1.74 HIV-1 DNA^+^ cells per 10^5^ cells) were only modestly above the limit of detection established for the assay. Importantly, neither HIV-1 p24, HIV-1 RNA or HIV-1 DNA was detectable in peripheral blood mononuclear cells (PBMCs) by repeated cell culture-based quantitative viral outgrowth assay nor by intact proviral DNA assay (Extended Data Table 2). Negative in vivo outgrowth assays using two different humanized mouse models confirmed the absence of replication-competent virus in the tested samples (Extended Data Fig. 2). Despite repeated in-depth reservoir assessments to determine whether or not the virus persisted and thus ensure the safest possible approach for ATI, the presence of residual replication-competent virus could not be completely ruled out^7,9,10^ and ATI ultimately remained the only way to prove HIV-1 cure^8,9^.

ART was discontinued 69 months after HSCT in November 2018, after careful consideration, and no antiretroviral agents were detected in four plasma samples collected after ATI (Fig. 1a). On cessation of ART, the patient remained without any clinical or laboratory signs of an acute retroviral syndrome. No rebound of plasma HIV-1 RNA occurred during ATI after 48 months in the absence of ART (Fig. 1a).

**Fig. 2:**
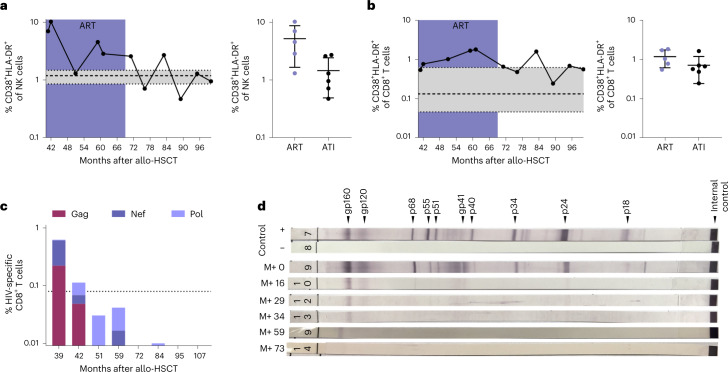
Waning HIV-1-specific cellular and humoral immune responses before and after ATI. **a**,**b**, NK cells (**a**) and CD8^+^ T cells (**b**) both showed decreasing and stable immune activation (coexpression of CD38 and HLA-DR). See also data from months 41–59 after HSCT for IciStem no. 19 as published in ref. ^8^. Data are shown as the mean with s.d. and a reference range from a healthy cohort (*n* = 8) in gray as the median with interquartile range (IQR). **c**, HIV-1-specific CD8^+^ T cell responses (production of IFNγ, TNFα, IL-2 and/or expression of CD107a) against HIV-1 Gag (burgundy), Nef (dark purple) and Pol (light purple) peptide pools waned after HSCT. See also the data from months 39 and 42 after HSCT for IciStem no. 19 as published in ref. ^8^. The dotted line represents the average background signal. **d**, Full-virus lysate immunoblot for antibodies against HIV-1 antigens revealed waning HIV-1-specific antibody responses after HSCT and prolonged weakening of gp160- and gp120-specific antibodies. M+, months after HSCT. Source data

**Extended Data Fig. 3:**
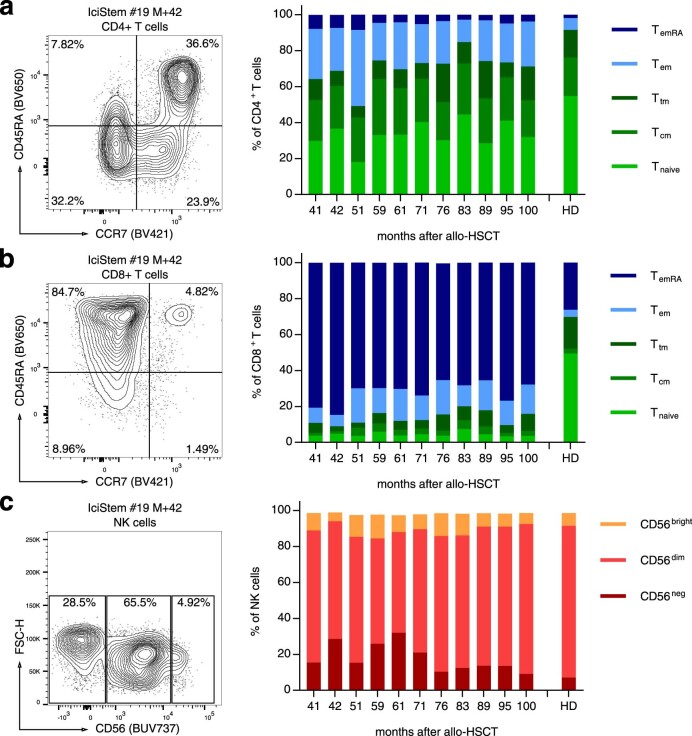
Immunological profiling before and after ATI. **a–b**, The distribution of memory subsets within the CD4+ and CD8+ T cell compartments showed markedly reduced frequencies of the early differentiated naïve T cells and elevated frequencies of the late differentiated T_em_ and T_emRA_ subsets. T_naive_, naïve T cells (CD45RA+ CCR7+); T_cm_, central memory T cells (CD45RA– CCR7+); T_tm_, transitional memory T cells (CD45RA– CCR7– CD27+); T_em_, effector memory T cells (CD45RA– CCR7– CD27–); T_emRA_, late effector memory T cells (CD45RA+ CCR7–); HD, healthy donors. **c**, Within the NK cell compartment, elevated frequencies of CD56^neg^ NK cells were observed. For all subsets, the mean frequency from a healthy cohort (n = 8) is given.

**Extended Data Fig. 4:**
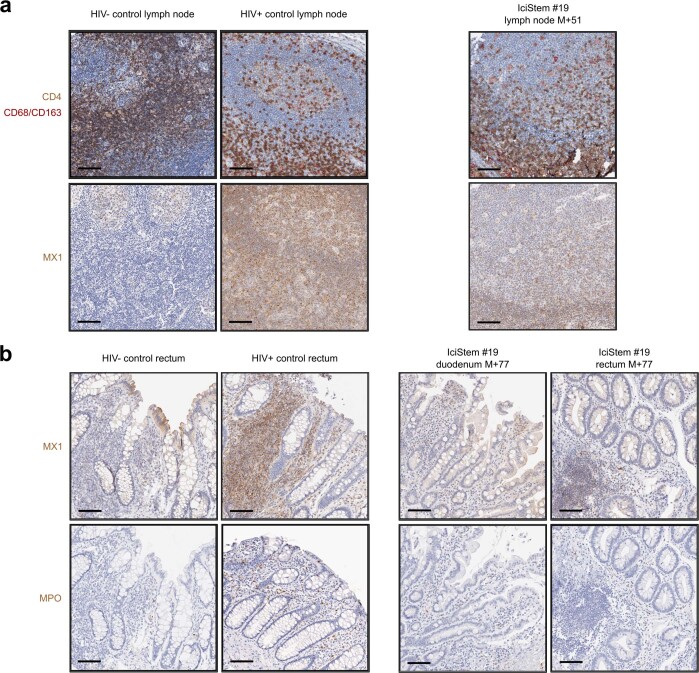
Immune reconstitution of the tissue-resident immune system after HSCT and tissue inflammation. **a**, The CD4+ T cell density is normal in lymphoid tissue. Slices were counterstained for macrophages (CD68/CD163) to allow the differentiation of CD4+ T cells from myeloid lineage cells by masking the faint CD4 expressed on these cells. MX1 (as a marker of type I IFN activation and inflammation) was not found to be strongly expressed in the lymphoid tissue slices. The slices of the lymphoid tissue are not sequential, but within 50 μm or less. MX1, Interferon-induced GTP-binding protein MX1; IFN, interferon. **b**, MX1 and MPO expression is not increased in gut tissue slices. MPO expression is indicative of PMN cell infiltration and thus of microbial translocation and gut barrier damage. For all immunohistochemical stainings of IciStem no. 19, the lymph node tissue was collected in month 51 after HSCT, and the gut biopsies were obtained in month 77 after HSCT. For comparison, tissue slices of HIV-1 negative and HIV-1 positive control patients are shown. The slices of the HIV-1 negative rectal tissue, duodenal and rectal tissue of the patient are not sequential, but within 50 μm or less. All slices were counterstained with hematoxylin. Scale bars represent 100 μm. MPO, myeloperoxidase; PMN, polymorphnuclear; HSCT, hematopoietic stem cell transplantation.

Extended immunological profiling before and after ATI demonstrated stable CD4^+^ T cell counts, absence of CCR5 expression (Extended Data Fig. 1c) and an immune status comparable to previous reports of people living with HIV (PLWH) after HSCT (with reduced naive T cell frequencies, elevated terminally differentiated effector memory T (T_EMRA_) cell frequencies and elevated frequencies of CD56^−^ natural killer (NK) cells)^8^ (Extended Data Fig. 3). The activation levels of the patient’s peripheral blood NK cells and cytotoxic CD8^+^ T cells after ATI were within the range observed in HIV-1-negative controls (Fig. 2a,b; see also previously published data in ref. ^8^). Moreover, the immune cell composition and, in particular, the CD4^+^ T cell density was normal in the investigated lymphoid tissue at the time of sampling (month 51 after HSCT) and in gut tissue samples obtained after ATI (month 77 after HSCT; Extended Data Fig. 4a). Additionally, there was no evidence of elevated inflammation in the lymphoid and gut tissue (measured by MX1 expression) or gut barrier damage (measured by MPO expression) by immunohistochemical staining (Extended Data Fig. 4b,c).

**Extended Data Fig. 5:**
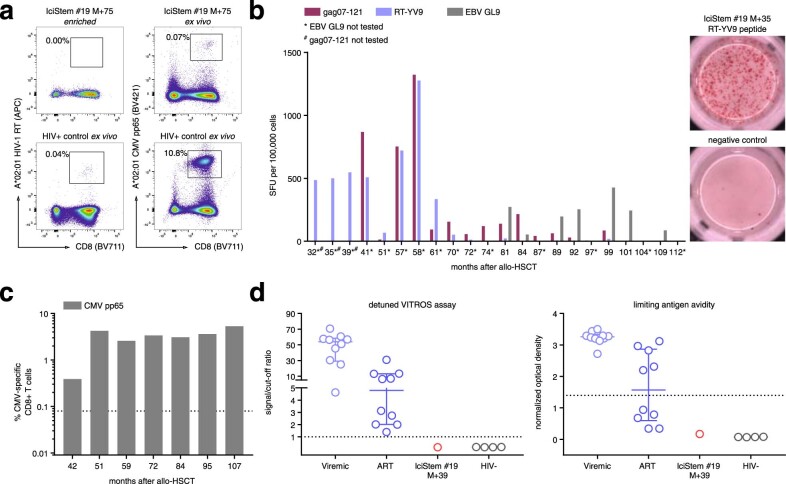
Cellular and humoral immune responses before and after ATI. **a**, No substantial frequencies of HIV-1 (RT-YV9; YQYMDDLYV) specific CD8+ T cells were found before or after ATI after magnetic bead MHC class I tetramer enrichment despite sustained CMV-specific responses ex vivo (performed at the University Medical Center Hamburg). Shown are representative plots from month 75 after HSCT. **b**, IFN-γ ELISpot assays showed waning HIV-1-specific T-cell responses of *in vitro* expanded polyclonal T-cell lines specific for the gag07-121 (KELYPLASLRSLFGN) and RT-YV9 (YQYMDDLYV) peptides despite maintained EBV-specific responses (performed at the Friedrich-Alexander University Erlangen-Nuremberg). IFN-γ, interferon-gamma; ELISpot, enzyme-linked immune absorbent spot; EBV, Epstein Barr Virus. **c**, Strong CMV-specific CD8+ T-cell responses were maintained before and after ATI as assessed by the production of IFN-γ, TNF-α, IL-2, and/or expression of CD107a in the ICS (performed at the Institut Pasteur Paris). The dotted line shows the average background signal. CMV, cytomegalovirus; ATI, analytical treatment interruption; ICS, intracellular cytokine staining; HSCT, hematopoietic stem cell transplantation. **d**, In month 39, the HIV-1-specific antibody levels and avidity were below the cut-off for PLWH (n = 10 viremic and n = 10 ART treated) and comparable to HIV-1 negative patients (n = 4) as measured by a detuned low-sensitive version of the HIV-1 VITROS assay and the limiting antigen avidity assay (performed at IrsiCaixa AIDS Research Institute Barcelona). Data are shown as median with IQR.

In month 39 after HSCT, HIV-1-specific CD8^+^ T cells were weakly detected on stimulation with overlapping peptide pools spanning HIV-1 Gag, Pol and Nef by intracellular cytokine staining (Fig. 2c; see also previously published data in ref. ^8^). However, the frequencies of HIV-1-specific T cells were substantially lower than previously observed for other PLWH^8,10^, further declined below threshold levels while still on ART and did not increase after ATI (Fig. 2c). Likewise, no significant T cell responses toward a set of 37 HIV-1 peptides known to be restricted by the patient’s major histocompatibility complex (MHC) class I molecules were detected before or after ATI as measured ex vivo by interferon-γ (IFNγ) enzyme-linked immunosorbent spot (ELISpot) (Supplementary Table 1) or MHC class I tetramer enrichment of an HLA-A*02-restricted reverse transcriptase epitope (RT-YV9) (Extended Data Fig. 5a). However, stimulation of PBMCs with the HLA-A*02-restricted RT-YV9 peptide and the gag-p6 peptide Gag07-121 elicited pronounced T cell responses in vitro after discontinuation of tacrolimus in month 56 that eventually decreased and were not detected in the most recent samples (Extended Data Fig. 5b). These short-term in vitro-expanded HIV-1-specific T cells were donor-derived because they carried the CCR5Δ32/Δ32 deletion (Extended Data Fig. 1b) suggesting antigen presentation to donor T cells in the peri-transplant period, despite continued ART^8^. In contrast, CMV-specific CD8^+^ T cells were strongly and consistently detected (Extended Data Fig. 5c), indicating that the waning of HIV-1-specific CD8^+^ T cells was probably due to the absence of stimulation by HIV antigens.

Immunoblot analyses of the HIV-1-specific antibody response paralleled the progressive loss of cellular HIV-1 reactivity: anti-gp120 and anti-gp160 antibodies showed the longest persistence (Fig. 2d), as reported previously^2,7^. At month 39 after HSCT, the peripheral blood HIV-1-specific antibody levels were below the cutoff for PLWH and comparable to HIV-1-negative individuals (Extended Data Fig. 5d). Because the maintenance of virus-specific responses is dependent on antigen exposure in chronic infection^11^, the extremely weak and waning HIV-1-specific T cell response and the decreasing levels of specific antibodies suggest that the HIV-1 reservoir capable of antigen production has been extremely depleted by the HSCT procedure and/or the graft versus HIV effect^1,2^.

Despite traces of HIV-1 DNA, there was no evidence of viral rebound or immunological correlates of antigen exposure. It is unclear whether the residual HIV-1 DNA signals stem exclusively from defective viral fragments or from an infinitesimally small pool of intact proviruses because proof for the absence of residual replication-competent virus was limited by the number of cells obtained and restricted accessibility of some anatomical compartments known to harbor the HIV-1 reservoir^9,10^. Therefore, it might be decisive that the patient was transplanted with a CCR5Δ32/Δ32 graft and that only an extremely low proportion (0.14%) of the proviral sequences retrieved before HSCT was characterized as possibly CXCR4-tropic, while potentially intact proviruses of the predominant R5-tropic population would not be able to propagate in this protective setting because of the absence of CCR5^+^ target cells^4^ (Extended Data Table 3).

The detailed observational characterization of single cases of HIV-1 cure after HSCT gives important insights but is anecdotal by nature and lacks the power of controlled prospective studies. Therefore, certain aspects of the cases published to date are of uncertain relevance for HIV-1 cure^6,7^, for example, underlying hematological malignancy, conditioning regimens or degree of GvHD as well as donor–recipient sex mismatch or the timing of ATI. However, two similarities are crucial for the outcome: all three patients achieved long-term remission after CCR5Δ32/Δ32 HSCT and harbored predominant R5-tropic virus strains. While CCR5Δ32/Δ32 HSCT cannot prevent the rebound of X4-tropic viruses^5^, this modification of the host’s immune system is a key component to prevent reservoir reseeding in PLWH with R5-tropic viruses requiring HSCT. Reservoir reduction during conditioning chemotherapy and immune-mediated HIV-1 clearance by donor cells (the ‘graft versus HIV effect’), and unspecific decay of latently infected cells driven by alloreactivity (GvHD; potentially further enhanced by DLI), might be additional, potential components that together led to the cure of HIV-1. However, it remains elusive to what extent reservoir depletion is necessary and contributes to HIV-1 cure in this and other cases^2,6–8^. One limitation of our study was the scarcity of samples available for detailed immunological and virological analysis directly before and after the HSCT.

Although HSCT using donors with a CCR5Δ32/Δ32 mutation is neither a low-risk nor an easily scalable procedure, its relevance to cure strategies is highlighted by recent reports of successful long-term HIV-1 remission after CCR5Δ32/Δ32-HSCT^7,12,13^. Expansion of this approach to introduce the CCR5Δ32 mutation into wild-type stem cell grafts using gene therapy in combination with new reservoir reduction strategies may hold the promise of an HIV-1 cure outside of life-threatening hematological malignancies. This third case of HIV-1 cure after allogeneic CCR5Δ32/Δ32 HSCT provides detailed information on the virological and immunological signature before and after ATI and generates valuable insights that will hopefully guide future cure strategies.

## Methods

### Ethics

The described individual (male, 53 years old as of 2022) was enrolled as patient no. 19 in the IciStem program at University Hospital Düsseldorf. ATI and examinations of the viral reservoir were performed after consultation of the ethics board of the Medical Faculty of the Heinrich Heine University Düsseldorf (official statement from 29 July 2016). Written informed consent was obtained from the patient for ATI and additionally performed immunological and virological studies (ethics board of the Medical Faculty of the Heinrich Heine University Düsseldorf no. 4261) in accordance with CAse REport (CARE) guidelines and the 2013 Declaration of Helsinki principles.

As controls for in situ hybridization assays and immunohistochemistry, a single lymph node and gut biopsies of one HIV-1-positive control (male, 56 years old) were collected in the ACS cohort, an ongoing, prospective multicenter acute HIV-1 infection cohort in Belgium, coordinated at the HIV Cure Research Center of Ghent University Hospital (ClinicalTrials.gov ID: NCT03449706) and approved by the ethics committee of Ghent University Hospital (no. BC-00812). The collection of rectum biopsies of one HIV-1-negative volunteer (male, 76 years old) undergoing screening colonoscopy at the HIV Cure Research Center at Ghent University Hospital was approved by the ethics committee of Ghent University Hospital (nos. BC-00812 and BC-11798). The lymph node of one HIV-1-negative individual (female, 41 years old) was provided by Knight BioLibrary (institutional review board-approved, no. IRB00004918) under full ethical approval by the Oregon Health & Science University institutional review board. Rectal tissue of one HIV-1-positive individual (male, 30 years old) for MPO staining was obtained from a study reported previously^14^.

For flow cytometry and MHC class I tetramer staining, eight HIV-1-negative controls (37.5% female, median age 25.5 years, range 21–28 years) and one HIV-1-positive control (male, 69 years old) were enrolled at the University Medical Center Hamburg-Eppendorf and were approved by the ethics board of the Hamburg Medical Association (nos. PV4780 and MC316/14).

All controls were enrolled on a voluntary basis, were not compensated and provided written informed consent, complying with the 2013 Declaration of Helsinki principles.

### HIV-1 RNA quantification and ultrasensitive viral load

Quantification of HIV-1 RNA in plasma and cerebrospinal fluid samples was carried out with clinical routine assays and three different automated platforms during the observation period between January 2011 and October 2022. Until December 2014, the Abbott RealTime HIV-1 assay was used on the Abbott m2000 system. From January 2015 to July 2021, HIV-1 RNA quantification was performed on the Roche CAP/CTM using the COBAS AmpliPrep/COBAS TaqMan HIV-1 Test v.2.0. Since August 2021, quantification has been performed on the Roche c6800 system using the COBAS HIV-1 Test Kit. All analyses were carried out according to the manufacturer’s instructions with a detection limit of 40 copies per ml (Abbott) and 20 copies per ml (Roche).

Ultrasensitive residual viremia was measured in 9 ml of plasma after ultracentrifugation at 170,000*g* at 4 °C for 30 min, followed by viral RNA extraction using the m2000sp Abbott RealTime HIV-1 Assay and laboratory-defined application software from the instrument^15^. HIV-1 RNA was quantified with a validated in-house calibration curve set with a limit of detection of 0.56 copies per ml.

### HIV-1 DNA quantification and droplet digital PCR

Clinical routine quantification of cellular HIV-1 DNA (proviral load) in PBMCs and buffy coat samples was performed with the Roche COBAS AmpliPrep/COBAS TaqMan HIV-1 Test v.2.0 on the Roche CAP/CTM platform or the Abbott RealTime HIV-1 Amplification Reagent Kit on the Abbott m2000rt Analyzer according to the manufacturer’s instructions. To exclude amplification of viral RNA during quantification of proviral DNA, cellular DNA was previously extracted automatically using the EZ1 DNA Blood 200 Kit (QIAGEN) via the EZ1 Advanced XL Platform or with the Abbott *m*Sample-Preparation System Kit on the Abbott Tecan m2000sp platform and used in equal amounts for quantification. The normalization of the detected HIV-1 DNA copies to the number of PBMCs was carried out by quantifying the housekeeping genes β-actin or β-globin.

Droplet digital PCR (ddPCR) was performed from different samples: PBMCs were isolated from blood samples. From there, CD4^+^ T bulk cells and four different subsets (naive T, central memory T, transitional memory T and effector memory T cells) were isolated by cell sorting. Gut biopsies (ileum, rectum and duodenum) were disaggregated by dithiothreitol/EDTA-based treatment for epithelial layer removal followed by overnight nonenzymatic disruption of the tissue to obtain lamina propria cell suspension^16^. CD45^+^ cells were subsequently purified by flow sorting using the BD FACS Aria cytometer (BD Biosciences). Follicular helper CD4^+^ memory T cells (CD3^+^CD4^+^CD45RA^+^PD1^+^CXCR5^+^) were sorted by flow cytometry from lymph node biopsy specimens using the BD FACS Aria cytometer.

In all cases, the isolated cells were lysed and viral DNA was quantified with ddPCR with HIV-1 LTR and Gag primers, as indicated in Supplementary Table 2 and as described previously^16–18^. Undetectable values are represented as a specific limit of detection adjusted to the number of cells used in each sample.

Quantification of intact provirus was assessed using lysed extracts of CD4^+^ T cells. Duplex ddPCR was performed using the packaging signal (Ψ) and nonhypermutated envelope gene (Env) primer and probe sets, according to the original proviral DNA assay protocol^19^.

### Viral coreceptor tropism analysis

Using PBMCs from day −7 before HSCT, the proviral load was determined and the Env region containing the complete V3 loop was amplified using an in-house protocol^20^ with primers as indicated in Supplementary Table 2. Genotypic coreceptor usage was predicted using the Geno2pheno [coreceptor] tool^21^ after Sanger sequencing.

Additionally, the amplified product was deep-sequenced using the Illumina MiSeq platform^22^. The sequence data are available via the NCBI GenBank database (accession nos. OP712709–OP713600). Genotypic coreceptor usage for all 17,701 reads (535 different V3 amino acid sequence variants) covering the whole V3 region was predicted using the Geno2pheno [454] tool^23^. In detail, for each variant, the false positive rate (FPR) of falsely classifying an R5-tropic virus as an X4-tropic virus was predicted; according to the determined cutoff of ≤3.5% FPR the single sequences were defined as R5- or X4-tropic. The total sample was considered to be X4-tropic if ≥2% of the viral population was scored as X4-tropic^24^. Of all 535 detected V3 variants, nine were selected for the phenotypic assay considering the following conditions to cover all variations: predicted R5, high FPR (nos.1–3); predicted R5, high FPR, high percentage (no. 4); predicted R5, low FPR, highest percentage (no. 5); predicted R5, low FPR (no. 6); predicted X4, low FPR, highest percentage (no. 7); predicted X4, low FPR (no. 8); predicted X4, lowest FPR (no. 9).

These nine sequences, which encode the full V3 region from nucleotide 7,110 to 7,217 (based on the HxB2 reference sequence), were cloned into the pHXB2-Δgp120-V3 vector^25^. The same vectors carrying either the gp120-V3 sequence of HxB2 (X4-tropic) or of BaL (R5-tropic) served as positive controls. The chimeric viruses were tested in cell culture with MT2 cells and the U373-MAGI-CCR5E (CD4^+^CCR5^+^CXCR4^−^) and U373-MAGI-CXCR4CEM (CD4^+^CCR5^−^CXCR4^+^) cell lines, all obtained from the HIV reagents program.

### Antiretroviral drug screening in plasma

Quantification of the following antiretroviral drug concentrations was performed using a liquid chromatography–tandem mass spectrometry method (4–5) validated according to Food and Drug Administration and European Medicines Agency guidelines on the TSQ Quantiva UltiMate 3000 RSLC: FTC, lamivudine (3TC), tenofovir, efavirenz, etravirine, nevirapine, rilpivirine, amprenavir, atazanavir, darunavir, indinavir, lopinavir, nelfinavir, ritonavir, saquinavir, tipranavir, dolutegravir (DTG), elvitegravir, raltegravir and maraviroc. Testing was performed by the Clinical Pharmacology Department of the University Medical Center in Utrecht at months 72, 75, 78 and 81 after HSCT (months 3, 6, 9 and 12 after ATI, respectively).

### HIV-1 clade B in situ hybridization (RNAscope/DNAscope)

HIV-1 in situ hybridization was performed as described previously with a set of HIV-1 clade B lineage-specific in situ hybridization riboprobes (cat. nos. 416111 and 425531, ACDBio) as described previously^26^. The target probes used bind to all HIV-1 genes and cover just over 4.2 kb of the genome. However, the assay cannot distinguish infected cells with intact HIV-1 genomes from cells with defective HIV-1 genomes. The FPR for this study was determined in two ways: First, using clinical specimens from IciStem no. 19, the HIV-1-specific probe was replaced with assay buffer while all other assay conditions remained constant. Second, the full assay, including the HIV-1-specific probe, was performed on an HIV-1-uninfected cell line control. The highest FPR across all controls determined the specific threshold (logarithm of the odds = 1.83 HIV-1 RNA^+^ cells per 10^5^ cells and 4.18 HIV-1 DNA^+^ cells per 10^5^ cells) for the assay.

### Immunohistochemistry

Immunohistochemistry was performed as described previously^26^. A multi-staining approach of CD4/CD68/CD163 to quantify CD4^+^ T cells was performed and allowed the intense staining of the macrophage/myeloid cell markers to mask the faint CD4 expressed on these cells and to distinctly identify CD4^+^ T cells from myeloid lineage cells as described previously. Heat-induced epitope retrieval was performed by heating sections in 0.01% citraconic anhydride containing 0.05% Tween-20 for Mx1 or citrate pH 6.0 for MPO in a pressure cooker (Biocare Medical) set at 122 °C or 125 °C for 30 s. Slides were incubated with blocking buffer and then with mouse anti-Mx1 monoclonal antibody (clone M143, 1:2,000; a gift from G. Kochs and the Department of Virology of the University of Freiburg) diluted in blocking buffer for 1 h at room temperature with or with rabbit anti-MPO (1:5,000, cat. no. A0398, Dako) diluted in blocking buffer overnight at 4 °C. After washing, endogenous peroxidases were blocked using 1.5% (v/v) H_2_O_2_ in Tri-buffered saline, pH 7.4, and the slides were incubated with rabbit or mouse Polink (−1 or −2) horseradish peroxidase and developed with ImmPACT 3,3′-diaminobenzidine (Vector Laboratories), according to the manufacturer’s recommendations. All slides were washed in tap water, counterstained with hematoxylin, mounted in Permount (Thermo Fisher Scientific) and scanned at high magnification (200×) using the AT2 Aperio System (Leica); all high-resolution images were stored in HALO (Indica Labs). Representative regions of interest were identified and high-resolution images were extracted from these whole-tissue scans using the HALO figure generation tool.

### Quantitative viral outgrowth assays

In months 36 and 37, 2 × 10^7^ PBMCs were isolated from whole-blood samples each and CD8^+^ T cells were removed (CD8-pluriBead). Activated PBMCs (phytohemagglutinin (PHA) and IL-2) were cultivated in bulk cultures directly or in co-culture with PBMCs (CD8^+^ T cell-depleted) of HIV-1-negative donors or in co-culture with TZM-bl HIV reporter cells (National Institutes of Health (NIH) HIV reagents program) for 4 weeks. Weekly, cell culture supernatants were tested using a sensitive reverse transcriptase assay (CAVIDI) and cellular DNA was analyzed by viral DNA PCR and lysates of TZM-bl cells for luciferase activity.

Leukapheresis was obtained 39 and 80 months after stem cell transplantation to measure infectious units per million CD4^+^ T cells. We set up a limiting dilution virus culture assay using the maximum cell number available (23 and 203 × 10^6^ CD4^+^ T cells at months 39 and 80, respectively). A limiting dilution virus culture assay was used to measure the replication-competent reservoir in purified CD4^+^ T cells as described previously^27^ with the minor modification of using three donors with three different activators (PHA 5 μg ml^−1^, PHA 0.5 μg ml^−1^ and OKT3) as blast cells to boost the virus. The frequency of infectious HIV-1 units per million CD4^+^ T cells was calculated based on the 14-d p24Gag readout and using IUPMStats v.1.0, based on the maximum likelihood method (http://silicianolab.johnshopkins.edu/).

### In vivo outgrowth assays in humanized mice

As an in vivo measure of residual replication-competent reservoir cells in the blood, we used two different humanized mouse models to transfer patient-derived CD4^+^ T cells, both at month 42 (refs. ^28,29^).

For the BALB/c Rag2^−/−^γc^−/−^ mice, the experimental protocols were performed according to the guidelines of the German Animal Protection Law and reviewed and approved by the Hamburg Medical Association (nos. OB-050/07 and WF-010/2011) and the Hamburg Free and Hanseatic City, Authority for Health and Consumer Protection (nos. 63/09 and 23/11). Mice were kept on a 12/12-h dark–light cycle (lights on at 6:00) in a temperature-controlled (21–23 °C) and humidity-controlled (45–65%) environment. Mice were transplanted with CD8^+^-depleted PBMCs according to ref. ^29^. For this, 6-week-old male or female mice were preconditioned with an intraperitoneal injection of 60–80 µl of Clophosome-A Clodronate Liposomes (FormuMax Scientific). Forty-eight hours later, mice were irradiated with a dose of 4 Gy (at 4 h before transplantation) from a cesium 137 source at 3.75 Gy min^−1^ (CSL-12; Conservatome). Subsequently, mice were transplanted with 1 × 10^6^ cells in 150 μl PBS containing 0.1% human AB serum (PAN Biotech) by intraperitoneal injection. At 7 weeks after transplantation, mice were subjected to end analyses. Human cell engraftment was verified by fluorescence-activated cell sorting (FACS) analysis of peripheral blood samples using retro-orbital sampling. Likewise, viremia was assayed by diluting cell-free mouse plasma with human serum (PAN Biotech) using the ultrasensitive (<20 HIV-1 RNA copies per ml) COBAS AmpliPrep/Cobas TaqMan HIV-1 Test v.2.0 (Hoffmann-La Roche). For the analysis of peripheral cells, 50–100 μl of blood was collected from the retro-orbital venous sinus into 100 μl of bleeding buffer (PBS plus 10 mM EDTA) and red blood cells were lysed by treatment with Red Blood Cell Lysing Buffer (Sigma-Aldrich). The white blood cell pellet was resuspended in FACS buffer (PBS containing 2% FCS and 2 mM EDTA) and stained with monoclonal antibodies. Single-cell suspensions of bone marrow, spleen and liver were prepared at necropsy by manual tissue dissection and filtering through a sterile 70-μm nylon mesh (BD Biosciences) for antibody staining and FACS analysis. Stained cells were analyzed in FACS buffer plus 1% paraformaldehyde using a FACSCanto (BD Biosciences) system with the BD FACSDiva software v.5.0.3 and FlowJo software v.7/9 (FlowJo LLC). To monitor human cell engraftment, retro-orbitally collected cells were stained with monoclonal antibodies raised against mouse CD45.2 (104), human CD45 (H130), CD4 (RPA-T4) and human CD3 (UCHT1) (eBioscience). Isotype antibodies and cells obtained from non-transplanted mice served as negative staining controls.

For NOD.Cg-*Prkdc*^*scid*^ *Il2rg*^*tm1Wjl*^/SzJ mice, procedures were performed according to protocol 8927, which was reviewed by the Animal Experimentation Ethics Committee of the University Hospital Germans Trias i Pujol (registered as B9900005) and approved by the Catalan government according to current national and European Union legislation on the protection of experimental animals. Mice were kept on a 12/12-h dark–light cycle (lights on at 8:00) in a temperature-controlled (20–24 °C) and humidity-controlled (40–70%) environment. Mice balanced in sex were supervised daily according to a strict protocol to ensure their welfare and were euthanized, if required, with isoflurane (inhalation excess). Experiments were followed as published previously^2,28^. Briefly, 80 million purified CD4^+^ T cells were infused in five 7-week-old mice (16 million per mouse). Whole-blood samples were collected at weeks 2, 4 and 6 when mice were euthanized. At every time point, plasma was used for the quantification of HIV-1 RNA. Whole blood was stained to calculate the human cell engraftment as the percentage of human CD45^+^ cells in the blood. Whole ACK-lysed blood cells and mechanically disaggregated murine spleen biopsies were used for HIV-1 DNA quantification by ddPCR with HIV-1 LTR and gag primers as described above.

### Immunophenotyping

All analyses were performed with thawed PBMCs as described previously^8^. A total of 1–2 × 10^6^ cells were used for the flow cytometry analyses and stained with Zombie NIR fixable viability dye (BioLegend) according to the manufacturer’s instructions and one of the following antibody panels. For the analysis of conventional T cells, fluorochrome-conjugated monoclonal antibodies targeting CCR5 (clone 2D7, BUV737, BD Biosciences), CD8 (clone RPA-T8, BV785), HLA-DR (clone L243, BV711), CD45RA (clone HI100, BV650), CXCR4 (clone 12G5, BV605), CCR7 (clone G043H7, BV421), CD4 (clone SK3, PerCP-Cy5.5), CD27 (clone M-T271, FITC, BD Biosciences), CD38 (clone HB-7, PE-Cy7), PD-1 (clone EH12.2H7, PE-Dazzle), CD25 (clone M-A251, PE), CD3 (clone UCHT1, Alexa Fluor 700), CD127 (clone A019D5, Alexa Fluor 647), CD14 (clone 63D3, APC-Cy7) and CD19 (clone HIB19, APC-Cy7). For the analysis of unconventional T cells and NK cells, fluorochrome-conjugated monoclonal antibodies targeting CD56 (clone NCAM16.2, BUV737, BD Biosciences), CD8 (clone RPA-T8, BV785), HLA-DR (clone L243, BV711), CD16 (clone 3G8, BV650), TCR Vα7.2 (clone 3C10, BV605), NKG2D (clone 1D11, BV421), CD4 (clone SK3, PerCP-Cy5.5), TCR Vδ2 (clone B6, FITC), CD38 (clone HB-7, PE-Cy7), CD161 (clone HP-3G10, PE-Dazzle), TCR γδ (clone 11F2, PE, BD Biosciences), CD3 (clone UCHT1, Alexa Fluor 700), CD127 (clone A019D5, Alexa Fluor 647), CD14 (clone 63D3, APC-Cy7) and CD19 (clone HIB19, APC-Cy7). Unless stated otherwise, antibodies were purchased from BioLegend. Before acquisition, cells were fixed with paraformaldehyde. All samples were acquired on an LSRFortessa analyzer (BD Biosciences) run by the FACSDiva software v.8 (BD Biosciences). Analysis of the flow cytometry data was performed in FlowJo v.10.7 for Windows. The gating strategy is depicted in Supplementary Fig. 2.

### Intracellular cytokine staining

T cell stimulation and intracellular cytokine staining were performed as described previously^8^. Briefly, thawed PBMCs were rested overnight at 37 °C and 5% CO_2_ in RPMI medium (RPMI 1640 supplemented with l-glutamine and antibiotics) with 20% heat-inactivated FCS. Cells were then incubated with overlapping peptide pools encompassing HIV-1 consensus subtype B Gag, Pol and Nef or HCMV pp65 (all obtained through the NIH AIDS Reagent Program, Division of AIDS, National Institute of Allergy and Infectious Diseases (NIAID), NIH, cat. nos. 12425, 12438, 12545 and 11549) at 2 μg ml^−1^ and anti-CD28/anti-CD49d costimulation (BD Bioscience) at 1 μl ml^−1^. No peptides were added to the negative controls. Phorbol myristate acetate (80 ng ml^−1^) together with ionomycin (1 μg ml^−1^; Sigma-Aldrich) was used as a positive control. Anti-CD107a V450 (BD Bioscience) was added to all conditions. Golgi stop (1 μg ml^−1^; BD Biosciences) and brefeldin A (10 μg ml^−1^; Sigma-Aldrich) were added 30 min after the start of all incubations. Cells were then stained with the LIVE/DEAD Fixable Aqua Dead Cell Stain kit (Thermo Fisher Scientific) and with anti-CD3 Alexa Fluor 700, anti-CD4 APC and anti-CD8a APC-Cy7 antibodies (BD Biosciences). Cytofix and Cytoperm (BD Biosciences) were used for cell permeabilization before staining for intracellular markers. Intracellular staining used anti-IFNγ PE-Cy7, IL-2 FITC and anti-tumor necrosis factor-α (TNFα) PE-CF594 (BD Biosciences). Cell staining was then measured with an LSRII flow cytometer (BD Biosciences). Results were analyzed with FlowJo v.10.5. Because of the limited number of circulating T cells at some time points after allo-HSCT, results were only considered when at least 1,000 CD8^+^ T cells could be analyzed and the number of positive events was at least 50% higher than the negative control. The background signal of the negative control was subtracted from the signal obtained with the peptide pools. The gating strategy is depicted in Supplementary Fig. 3.

### MHC class I tetramer staining

MHC class I tetramer staining was performed as described previously^30^ using PBMCs from months 51, 61, 75 and 87 after HSCT. Thawed PBMCs were stained with APC-A*02:01 HIV-1 RT-YV9 (YQYMDDLYV) and additionally BV421-A*02:01 CMV pp65 (NLVPMVATV) in the unenriched fractions, both obtained from the NIH Tetramer Core Facility. Tetramer enrichment was performed with anti-APC microbeads applying magnetic-activated cell sorting technology (Miltenyi Biotec) according to the manufacturer’s protocol. Cells were stained with Zombie NIR fixable viability dye (BioLegend) according to the manufacturer’s instructions and fluorochrome-conjugated monoclonal antibody targeting CD45RA (clone HI100, BUV737, BD Biosciences), CD38 (clone HB-7, BUV395, BD Biosciences), Tim-3 (clone F38-2E2, BV785), CD8 (clone RPA-T8, BV711), PD-1 (clone EH12.2H7, BV650), TIGIT (clone A15153G, BV605), CD3 (clone UCHT1, BV510), CD39 (clone A1, BV421; only enriched fractions), CD127 (clone A019D5, PerCP-Cy5.5), CD27 (clone M-T271, FITC, BD Biosciences), CCR7 (clone G043H7, PE-Cy7), HLA-DR (clone L243, Alexa Fluor 700), CD14 (clone 63D3, APC-Cy7) and CD19 (clone HIB19, APC-Cy7). Before acquisition, cells were fixed with paraformaldehyde. All samples were acquired on an LSRFortessa analyzer (BD Biosciences) run by the FACSDiva software v.8 (BD Biosciences). The analysis of the flow cytometry data was performed in FlowJo v.10.7 for Windows.

### Ex vivo detection of HIV-1-specific T cells by IFNγ ELISpot

In the IFNγ ELISpot assay, 2 × 10^5^ PBMCs per well were incubated for 40 h with HIV-1 peptides corresponding to known cytotoxic T lymphocyte (CTL) epitopes restricted by the patient’s HLA alleles (Supplementary Table 1) at a final concentration of 6 μg ml^−1^. Concanavalin A and PHA (Sigma-Aldrich) served as positive controls. Peptides were either purchased from EMC Microcollections or kindly provided by the NIH AIDS Research and Reference Reagent Program or by the AIDS Reagent Project of the Medical Research Council and the European Community EVA Programme.

### In vitro expansion and analysis of HIV-1-specific T cells

Five million PBMCs each were stimulated with peptides corresponding to known CTL epitopes restricted by the patient’s HLA alleles at a final concentration of 6 μg ml^−1^ in 1 ml of RPMI 1640 medium (Gibco) containing 10% heat-inactivated FCS (Gibco), 1% l-glutamine (2 mmol l^−1^), penicillin (100 U ml^−1^), streptomycin (100 μg ml^−1^), HEPES (10 mmol l^−1^) (Merck) and 10 U ml^−1^ recombinant IL-2 (Proleukin). After 5–32 d, outgrowing cells were analyzed by IFNγ ELISpot assay for recognition of the peptides at a cell concentration of 1 × 10^5^ cells per well. ELISpot assays were conducted using R5AB medium consisting of RPMI 1640 medium with supplements and 5% heat-inactivated human AB serum (Sigma-Aldrich).

### Detection of CCR5-32-bp deletion

Cellular DNA was isolated from PBMC and T cell expansions using the NucleoSpin Blood QuickPure kit (Macherey-Nagel) according to the manufacturer’s instructions. Aliquots of DNA were amplified using Taq polymerase (Invitrogen) and 40 cycles (94 °C, 40 s; 56 °C, 50 s; 72 °C, 60 s) in a volume of 25 μl using the primers CCR5-5 760 (5′-CTGCAGCTCTCATTTTCC-3′) and CCR5-3 982 (5′-TCAGGAGAAGGACAATGTTG-3′). By separating the reaction products (8 μl) on 2% metaphore agarose (FMC) with MIDORI green, the presence of the 32-bp deletion could be visualized. As an additional control for the method, the visualized amplification product of the RT-YV9-specific CTL line was cut out from the agarose gel and analyzed with Dye Terminator Cycle Sequencing (PE, Applied Biosystems). The PCR products were sequenced on both strands using the same oligonucleotides as primers. PBMC and B cell lines from CCR5 wild-type, Δ32 heterozygous and Δ32 homozygous individuals served as controls.

### Quantification of anti-HIV-1 antibodies

Specific anti-HIV-1 antibodies in plasma samples were measured using a qualitative full-virus lysate immunoblot assay (New LAV Blot, Bio-Rad Laboratories) based on an indirect enzyme-linked immunosorbent assay technique. Ready-to-use nitrocellulose strips contained all inactivated HIV-1 constituent proteins separated by their molecular weights and an internal anti-IgG control. In contrast to recombinant immunoblots, the position of the antigen bands can vary between lots. Additionally, the quantitative standard VITROS anti-HIV-1 immunoassay, a low-sensitivity version of the VITROS anti-HIV-1 immunoassay (Ortho Clinical Diagnostics), and the limiting antigen-avidity assay were performed as described previously^31^ to assess anti-HIV-1 antibody levels.

### Statistics and reproducibility

Graphs were created and statistical analyses were performed using Prism 7 for Windows (GraphPad Software). For statistical testing, a nonparametric two-tailed Mann–Whitney *U*-test was performed. Reference values from healthy controls are displayed as the median with IQR. Sex bias could not be considered because this study focused on one specific individual.

Biological replicates (that is, samples at different time points) were measured in all experiments except in situ hybridization, immunohistochemistry and CCR5-PCR given the limited clinical material, tropism analysis given lack of viral sequences due to undetectable (pro)viral loads and mVOAs given ethical concerns and limited clinical material. Technical replicates (that is, repeated measuring of the same sample) were measured as follows: for clinical viral load testing at each time point, two separate plasma samples were collected and measured independently from one another with similar results; for ddPCR, technical replicates were measured as indicated in Extended Data Table 2 with similar results (any divergent results are reported due to extremely rare abundance of HIV-1 DNA traces); for mVOAs, *n* = 2 and *n* = 5 mice were transplanted with the tested cells and generated similar results; the in situ hybridization, immunohistochemistry and flow cytometry experiments were not technically replicated given the limited clinical material; CCR5-PCR was done from PBMCs ex vivo and two different T cell expansions with similar results; most HIV-1-specific antibody immunoblots were measured once due to limited clinical material. The conclusiveness comes from coherent results due to periodic measurements.

### Reporting summary

Further information on research design is available in the Nature Portfolio Reporting Summary linked to this article.

## Online content

Any methods, additional references, Nature Portfolio reporting summaries, source data, extended data, supplementary information, acknowledgements, peer review information; details of author contributions and competing interests; and statements of data and code availability are available at 10.1038/s41591-023-02213-x.

### Supplementary information

Supplementary Information Supplementary Figs. 1–3 and Tables 1 and 2.

Reporting Summary

### Source data

Source Data Fig. 2 Full immunoblot images.

Source Data Extended Data Fig. 1 Full gel electrophoresis.

## Figures and Tables

**Extended Data Table 1:**
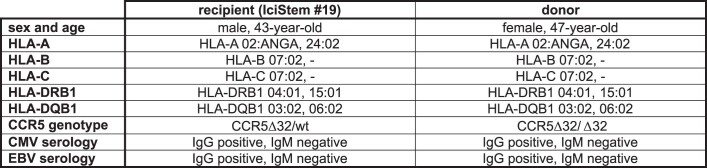
Donor characteristics and HLA-matching

Compilation of the sex and age of the recipient and donor at HSCT, their HLA and CCR5 genotype, and CMV and EBV serologies. CMV, cytomegalovirus; EBV, Epstein–Barr Virus.

**Extended Data Table 2:**
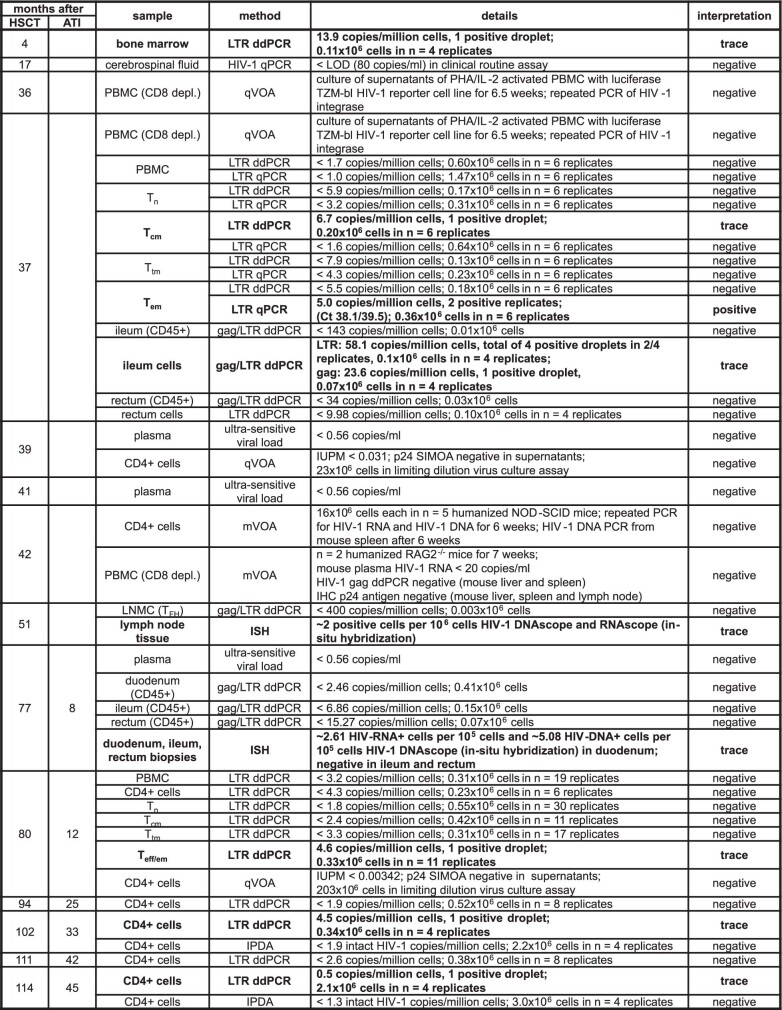
HIV-1 reservoir analyses

HIV-1 reservoir was assessed in peripheral blood, bone marrow, lymph node and gut tissue. For quantification of HIV-1 DNA in peripheral T cell subsets and tissue-derived cells, HIV-1 LTR ddPCR was applied. In 119 negative controls (*n* = 83 no template controls and *n* = 36 donor PBMC controls) used in 20 experiments for the LTR ddPCR assays of the peripheral blood, only one positive droplet was detected in a no-template control. Additionally, ddPCR with gag primers and qPCR approaches were used at certain time points. In gut- and lymph node-derived tissue sections, in situ hybridization was used to detect HIV-1 DNA and RNA. Quantitative and two different murine viral outgrowth assays were performed to exclude replication-competent virus isolates. For individual time points, ultrasensitive residual HIV-1 RNA in plasma was quantified. ATI, analytical treatment interruption; Ct, cycle threshold; ddPCR, droplet digital polymerase chain reaction; HSCT, hematopoietic stem cell transplantation; IHC, immunohistochemistry; IL-2, interleukin-2; IPDA, intact proviral DNA assay; ISH, in situ hybridization; IUPM, infectious units per million; LNMC, lymph node mononuclear cell; LTR, long terminal repeat; mVOA, murine viral outgrowth assay; PBMC, peripheral blood mononuclear cell; PHA, phytohemagglutinin; qPCR, quantitative polymerase chain reaction; qVOA, quantitative viral outgrowth assay; SIMOA, single molecule array; T_n_, naive T cell; T_cm_, central memory T cell; T_tm_, transitional memory T cell; T_em_, effector memory T cell; T_FH_, follicular helper T cell.

**Extended Data Table 3:**
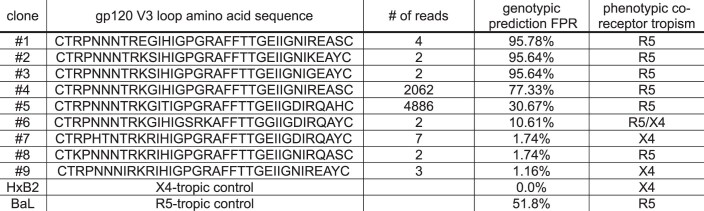
Phenotypic viral coreceptor tropism analysis for selected variants yielded from deep sequencing

Genotypic coreceptor usage was predicted before HSCT using the geno2pheno[454] tool after deep sequencing of the HIV-1 envelope V3 loop (nucleotide 7110–7217 based on the HxB2 reference sequence). In a total of *n* = 17,701 reads, 0.14% were predicted to be X4-tropic viral sequences. Results are expressed as the probability of classifying an R5-tropic virus falsely as a X4-tropic virus (cutoff 3.5%; FPR ≤ 3.5%, X4-tropic; FPR > 3.5%, R5-tropic). For the phenotypic coreceptor analyses, the nine representative amino acid sequences of the V3 loop shown in the table were cloned into a pHXB2-Δgp120-V3 vector and tested in T cell culture with MT2 cells and the U373-MAGI-CCR5E (CD4^+^CCR5^+^CXCR4^−^) and U373-MAGI-CXCR4CEM (CD4^+^CCR5^−^CXCR4^+^) cell lines. FPR, false positive rate; R5, CCR5-tropic virus; X4, CXCR4-tropic virus.

## Data Availability

Figures 1 and 2 and Extended Data Figs. 1–5 contain raw data. The HIV-1 sequence data are available via the NCBI GenBank database (accession nos. OP712709–OP713600). The other data that support the findings of this study are available upon request from the corresponding authors B.-E.O.J. and J.S.z.W. within the data protection constraints of the written informed consent signed by the study participants (that is, pseudonymized data only); they will be made available within 6 weeks. The data are not publicly available because they contain information that could compromise the participants’ privacy. Source data are provided with this paper.
