# Supplementary material for: In-depth virological and immunological characterization of HIV-1 cure after CCR5Δ32/Δ32 allogeneic hematopoietic stem cell transplantation
Source: Nat Med. 2023 Feb 20;29(3):583–7. doi: 10.1038/s41591-023-02213-x (PMC10033413; doi:10.1038/s41591-023-02213-x)
Supplement: Supplementary file 1 — Supplementary Figs. 1–3 and Tables 1 and 2. [file 41591_2023_2213_MOESM1_ESM.pdf]

nature medicine

Brief Communication

https://doi.org/10.1038/s41591-023-02213-x

In-depth virological and immunological characterization of HIV-1 cure after CCR5Δ32/Δ32 allogeneic hematopoietic stem cell transplantation

In the format provided by the authors and unedited

a

mouse spleen

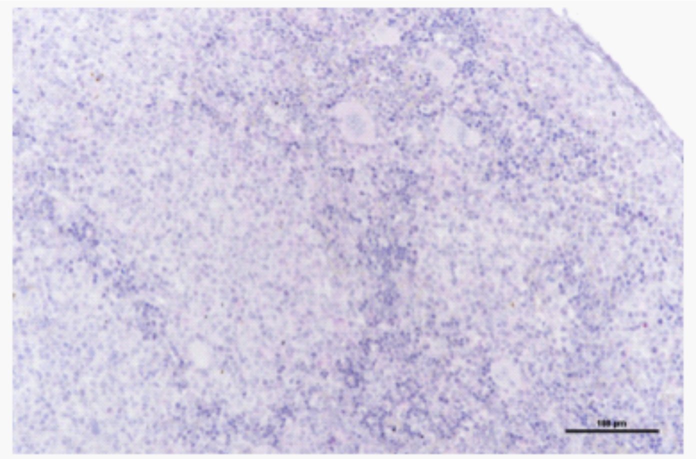

b

mouse liver

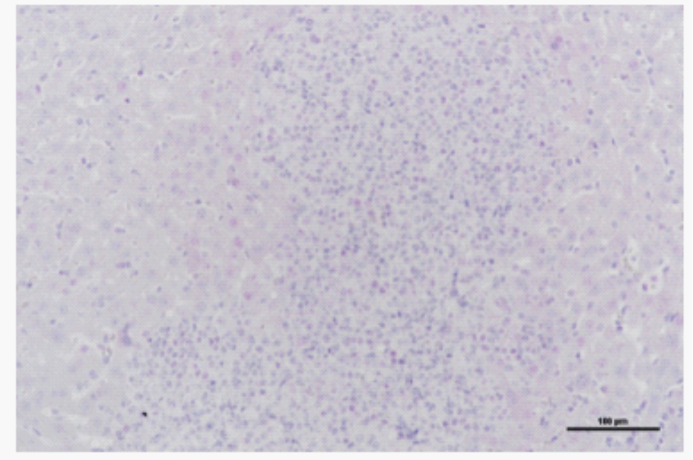

c

mouse lymph node

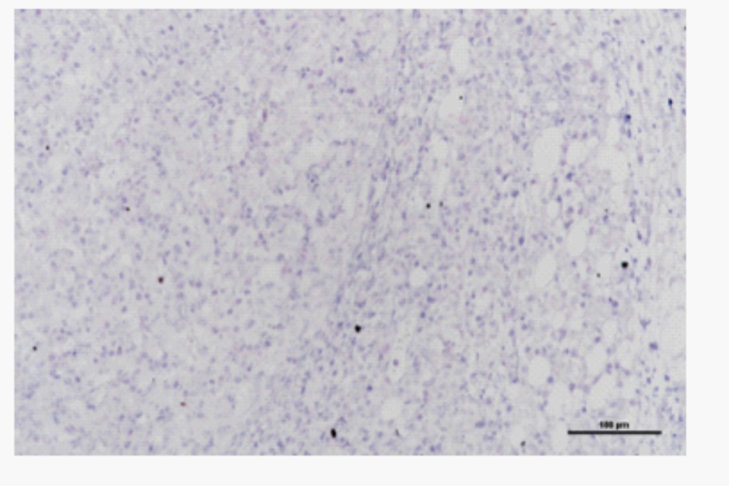

Supplementary Figure 1. HIV-1 p24 immunohistochemistry of mouse lymph node, liver and spleen.
a-c, In vivo viral rescue assay in n = 2 Balb/c Rag2$^{-/-}\gamma c^{-/-}$ mice showed negative results in the immunohistochemical stainings for HIV-1 p24 Ag in the lymph nodes, livers and spleens.

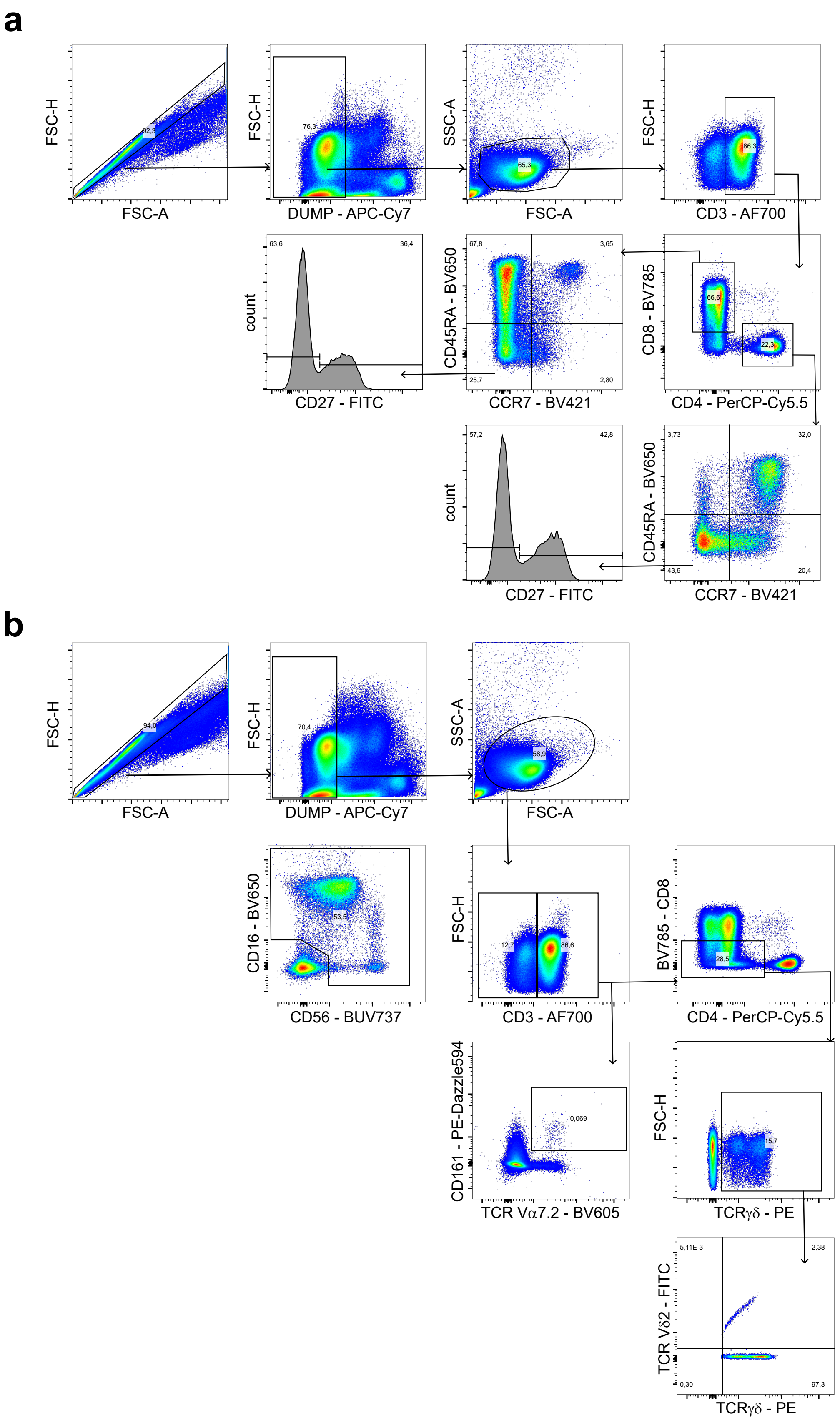

Supplementary Figure 2. Gating strategy for immunophenotyping.

a, Conventional T cells were identified by CD3 expression on single, living, CD14-, CD19- lymphocytes. T cells were then subdivided in CD4+CD8- T helper cells and CD4- CD8+ cytotoxic T cells. Memory subsets were differentiated in T_n (CD45RA+ CCR7+), T_{cm} (CD45RA- CCR7+), T_{tm} (CD45RA- CCR7- CD27+), T_{em} (CD45RA- CCR7- CD27-) and T_{emRA} (CD45RA+ CCR7-). **b**, MAIT cells were defined as TCR Vα7.2+ CD161+ T cells and γδ-T cells as TCRγδ+ CD4- CD8- T cells.γδ-T cells were subdivided in Vδ2+ and Vδ2- subsets. NK cells were **defined as CD16+ and/or** CD56+ non-T cells (CD3-).

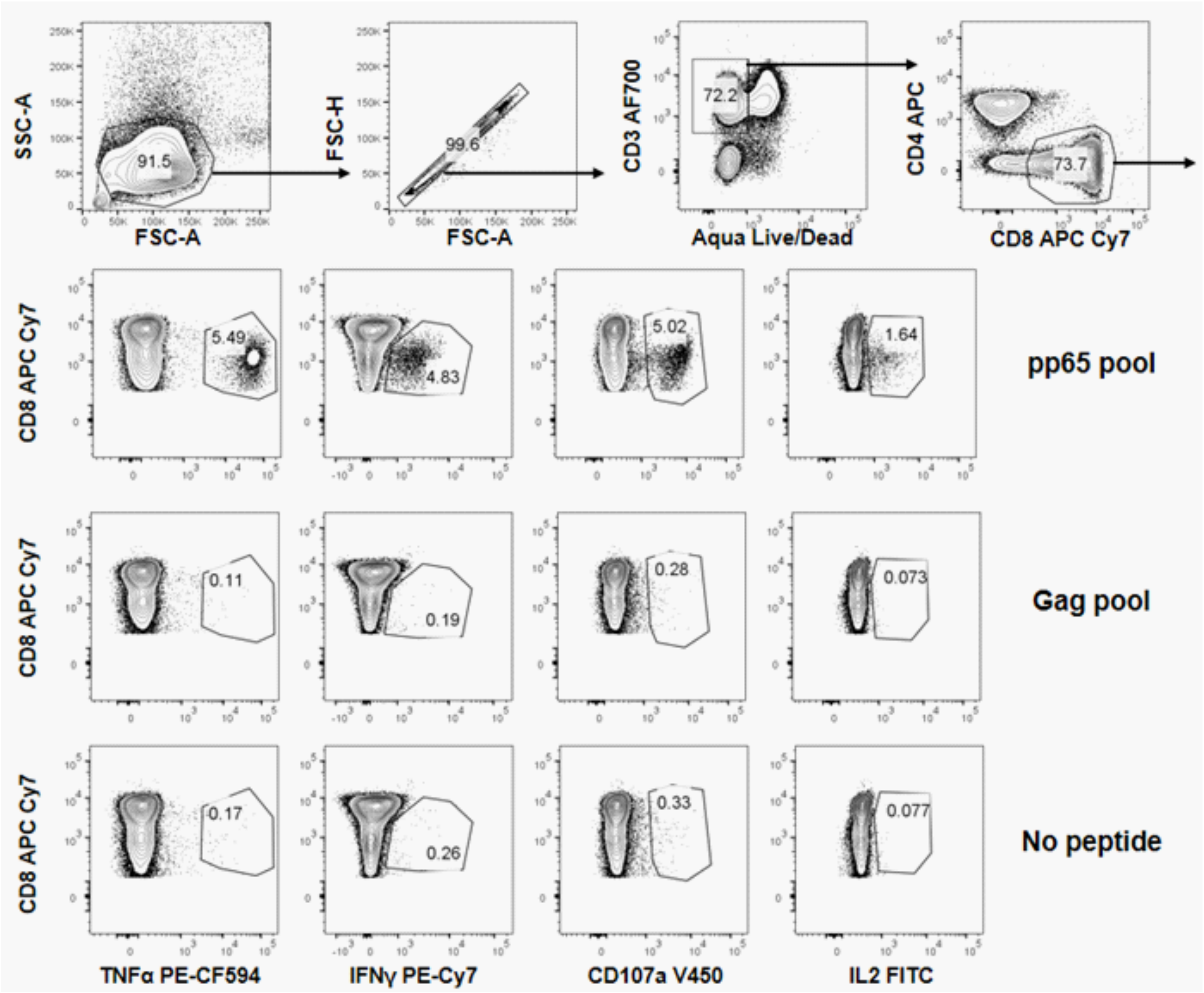

Supplementary Figure 3. Gating strategy for intracellular cytokine staining.
T cells were identified by CD3 expression on single living lymphocytes. Cytokine expression and CD107a expression was assessed for CD4-CD8+ cytotoxic T cells after stimulation with CMV pp65 or HIV-1 peptide pools. No addition of peptides served as negative controls.

			Months after HSCT:																		
**peptides/mitogens**	**peptide sequence**	**HLA restriction**	**32**	**35**	**59**	**61**	**70**	**72**	**74**	**77**	**79**	**81**	**84**	**87**	**89**	**92**	**97**	**99**	**101**	**104**	**111**
no peptide (negative control)			0	0	0	0	1	0	0	1	0	0	0	0	0	0	0	0	0	0	0
RT YV9	**YQYMDDLYV**	A2	4	0	0	0	6	0	0	0	0	0	0	0	0	1	2	3	0	0	0
RT VL9	**VIYQYMDDL**	A2	1	n.d.	0	1	1	n.d.	0	1	0	0	0	1	1	0	0	0	1	0	0
RT IV9	**ILKEPVHGV**	A2	1	n.d.	0	0	0	n.d.	0	1	0	0	0	0	0	0	0	0	0	0	0
p17 SL9	**SLYNTVATL**	A2	1	0	0	3	3	n.d.	0	1	0	0	0	0	0	0	3	3	0	0	0
Protease-KI10-M	**KMIGGIGGFI**	A2	3	n.d.	0	0	n.d.	n.d.	0	0	0	0	0	0	0	0	n.d.	n.d.	n.d.	n.d.	n.d.
pol07-66	VT**VLDVGDAYFSV**PL	A2	0	n.d.	0	0	0	0	0	1	0	0	0	0	0	0	0	0	0	0	0
pol07-78	KG**SPAIFQSSM**TKIL	B7	n.d.	n.d.	0	0	0	1	1	1	0	0	0	0	0	0	0	0	0	0	0
gag07-121	KE**LYPLASLRSLF**GN	A24,B7	n.d.	n.d.	0	0	1	1	1	1	0	0	0	0	0	0	0	0	0	0	0
gag07-121-YL9	**YPLASLRSL**	B7	n.d.	n.d.	6	0	6	0	0	1	0	0	0	0	0	0	1	1	0	0	0
gag07-121-LL10	**LYPLASLRSL**	A24,B7	n.d.	n.d.	0	0	0	0	0	0	0	0	0	0	0	0	0	0	0	0	0
gag07-121 LF11	**LYPLASLRSLF**	A24,B7	n.d.	n.d.	n.d.	n.d.	n.d.	n.d.	n.d.	n.d.	n.d.	n.d.	0	0	n.d.	0	0	0	0	0	0
gag07-7	GKK**KYKLKHIVW**ASR	A24	n.d.	n.d.	0	1	1	0	0	1	0	0	0	0	0	n.d.	0	0	0	0	0
gag07-38	P**RTLNAWVKV**VEEKA	A2	n.d.	n.d.	0	0	0	0	0	3	0	0	0	0	0	0	0	0	0	0	0
gag07-65	PVG**EIYKRWIIL**GLN	A24	n.d.	n.d.	0	0	0	0	0	1	0	0	0	0	0	0	0	0	0	0	0
gag07-68	GLNKIV**RMYSPTSIL**	A2	n.d.	n.d.	0	1	1	0	0	0	0	0	0	0	0	0	0	0	0	0	0
gag07-69	IV**RMYSPTSIL**DIRQ	A2,A24	n.d.	n.d.	0	0	0	0	0	0	0	0	0	0	0	0	0	0	0	0	0
gag07-73	PK**EPFRDYVDRF**YKT	A2	n.d.	n.d.	0	1	1	0	0	0	0	0	0	0	0	0	0	1	1	1	0
gag07-88	ACQGVG**GPGHKARVL**	B7	n.d.	n.d.	0	0	0	0	0	0	0	0	0	0	0	0	0	0	0	0	0
gag07-91	**RVLAEAMSQV**TNSAT	A2	n.d.	n.d.	0	0	0	0	0	0	0	0	0	0	0	0	0	0	0	0	0
p24-2	VH**QAISPRTLNAWV**KVVEEK	A2,B7,C7	4	n.d.	0	0	0	0	0	1	0	0	0	0	0	0	0	0	0	0	0
p24-5	SALSEG**ATPQDLNTM**LNTVG	B7,	n.d.	n.d.	0	1	1	1	1	2	0	0	0	0	0	0	0	0	0	0	0
Nef7-V1	**FPVRPQVPL**	B7	1	n.d.	0	0	0	0	0	1	0	0	0	0	0	0	0	0	0	0	0
Nef7-V2	**FPVTPQVPL**	B7	14	0	0	0	0	0	0	1	0	0	0	0	0	0	0	0	0	0	0
Nef-RY10	**RYPLTFGWCY**	A24	1	n.d.	0	1	1	0	0	2	0	0	0	0	0	0	0	0	0	0	0
Nef-TM9	**TPQVPLRPM**	B7	2	n.d.	0	2	2	1	0	0	0	0	0	0	0	0	0	1	0	0	0
Nef11-T3	**RQDILDLWIY**	C7	2	n.d.	0	0	0	1	0	1	0	0	0	0	0	0	0	0	0	0	0
Nef-AL9	**AFHHVAREL**	A2	n.d.	n.d.	0	0	0	0	0	1	0	0	0	0	0	0	0	0	0	0	0
Nef-DV9	**DSRLAFHHV**	A24	n.d.	n.d.	0	0	0	4	0	0	0	0	2	0	0	0	1	1	1	1	0
Nef13	PDWQ**NYTPGPGVRYPL**TFGW	A24,B7	n.d.	n.d.	0	0	0	2	0	1	0	0	0	0	0	0	0	0	0	0	0
Nef-TL10	**TPGPGVRYPL**	B7	n.d.	n.d.	0	0	0	0	0	2	0	0	0	0	0	0	0	0	0	0	0
Nef-QK10	**QVPLRPMTYK**	A3	n.d.	n.d.	0	0	0	0	0	0	0	0	0	0	0	0	0	0	0	0	0
Nef-VY8	**VPLRPMTY**	B7	n.d.	n.d.	0	0	0	0	0	1	0	0	0	0	0	0	1	1	1	1	2
Nef-Mu12	**RPMTYKAAV**	B7	n.d.	n.d.	0	0	0	0	0	0	0	0	0	0	0	0	0	0	0	0	0
Nef-Mu15	**RPMTYKAAL**	B7	1	n.d.	0	0	0	0	0	1	0	0	0	0	1	0	0	0	0	0	0
Env07-8	AAE**KLWVTVYYGVPV**	A2	n.d.	n.d.	0	0	0	0	0	1	0	0	0	0	0	0	0	0	0	0	0
Env07-9	LW**VTVYYGVPVWKEA**	A2, C7	n.d.	n.d.	n.d.	nd	n.d.	0	0	1	0	0	0	0	0	0	0	0	0	0	0
Env07-75	T**RPNNNTRKS**IHIGP	B7	n.d.	n.d.	0	0	0	0	0	0	0	0	0	0	0	0	0	0	0	0	0
JC Virus SL9	**SITEVECFL**	A2	n.d.	n.d.	0	0	0	0	0	1	0	0	0	0	0	0	0	0	0	0	0
JC Virus IL9	**ILMWEAVTL**	A2	n.d.	n.d.	0	1	0	0	0	0	0	0	0	0	0	0	0	0	0	0	0
EBV GL9	**GLCTLVAML**	A2	n.d.	n.d.	6	2	6	6	6	6	4	10	0	3	10	7	0	21	15	4	6
Influenza IMP-GL9	**GILGFVFTL**	A2	n.d.	n.d.	0	0	0	1	4	1	1	1	1	0	0	1	0	0	0	1	0
positive control: ConA			n.d.	n.d.	87	509	546	731	587	605	583	523	117	140	400	234	462	617	407	242	291
positive control: PHA			234	206	238	497	540	637	489	573	509	287	310	55	310	423	629	465	705	119	444

Supplementary Table 1. ***Ex vivo*** **analysis of HIV-1-specific T-cells in an IFN-γ ELISpot assay.**
Shown are Spot forming units (SFUs) per 200,000 freshly isolated PBMC in a 40 hours IFN-γ ELISpot assay.SFUs are given as mean of duplicates. n.d. = not done. SFU values considered as positive if > 5 SFU/200,000 PBMC. The mitogens PHA and ConA served as positive controls. Peptide sequences: amino acids in bold are contained in known T-cell epitopes. In addition to peptides corresponding to known HIV-1-specific T-cell epitopes, further peptides corresponding to HLA-A2-restricted epitopes derived from JC Virus, EBV and influenza were used.

a

for ddPCR from bone marrow and ileum/rectum cells	
LTR forward	5'-GCCTCAATAAAGCTTGCC-3'
LTR reverse	5'-GGCGCCACTGCTAGAGATTTT-3'
LTR probe	5'-AAGTRGTGTGTGCCC-3'
gag sense	5'-TCAGCCCAGAAGTAATACCCATGT-3'
gag antisense	5'-CACTGTGTTTAGCATGGTGTTT-3'
gag probe	5'-ATTATCAGAAGGAGCCACCCCACAAGA-3'
**for ddPCR from CD45+ LPL from gut biopsies and T follicular helper cells**	
LTR forward	5'-GTTCGGGCGCCACTGCTAG-3'
LTR reverse	5'-TTAAGCCTCAATAAAGCTTGCC-3'
LTR probe	5'-CCAGAGTCACACAACAGACGGGCA-3'
gag forward	5'-CATGTTTTCAGCATTATCAGAAGGA-3'
gag reverse	5'-TGCTTGATGTCCCCCCACT-3'
gag probe	5'-CCACCCCACAAGATTTAAACACCATGCTAA-3'
**for ddPCR from PBMC and T-cell subsets**	
LTR forward	5'-AAGCCTCAATAAAGCTTGCCTTGA-3'
LTR reverse 1	5'-GAGGGATCTCTAGTTACCAGAGTCACA-3'
LTR reverse 2	5'-GAGGGATCTCTAGTTACCAGAGTCCTA-3'
LTR probe	5'-TAGTGTGTGCCCGTCTG-3'

b

for envelope V3 RT-PCR	
forward	5'-CAAAGCCTAAAGCCATGTGTAAA-3'
reverse	5'-AGTGCTTCCTGCTGCTCCTAAGAACCC-3'
forward (nested)	5'-GTCCAAAGGTATCCTTTGAGCCAATTC-3'
reverse (nested)	5'-CACCACTCTTCTCTTTGCCTTGGTGGGTGC-3'

Supplementary Table 2. Primer sequences for V3 loop amplification and HIV-1 ddPCR.
a, Primer sequences for HIV-1 provirus quantification by ddPCR from different materials as indicated in Extended Data Table 2. **b**, Primer sequences for HIV-1 envelope V3 loop nested RT-PCR for tropism analysis.
