# Supplementary material for: In-depth virological and immunological characterization of HIV-1 cure after CCR5Δ32/Δ32 allogeneic hematopoietic stem cell transplantation
Source: Nat Med. 2023 Feb 20;29(3):583–7. doi: 10.1038/s41591-023-02213-x (PMC10033413; doi:10.1038/s41591-023-02213-x)
Supplement: Supplementary file 2 — Reporting Summary [file 41591_2023_2213_MOESM2_ESM.pdf]

nature portfolio

Corresponding author(s): Dr. Björn-Erik Ole Jensen
Prof. Dr. Julian Schulze zur Wiesch

Last updated by author(s): Dec 20, 2022

Reporting Summary

Nature Portfolio wishes to improve the reproducibility of the work that we publish. This form provides structure for consistency and transparency in reporting. For further information on Nature Portfolio policies, see our Editorial Policies and the Editorial Policy Checklist.

Statistics

For all statistical analyses, confirm that the following items are present in the figure legend, table legend, main text, or Methods section.

n/a	Confirmed	
☐	☒	The exact sample size (*n*) for each experimental group/condition, given as a discrete number and unit of measurement
☐	☒	A statement on whether measurements were taken from distinct samples or whether the same sample was measured repeatedly
☐	☒	The statistical test(s) used AND whether they are one- or two-sided<br>*Only common tests should be described solely by name; describe more complex techniques in the Methods section.*
☒	☐	A description of all covariates tested
☒	☐	A description of any assumptions or corrections, such as tests of normality and adjustment for multiple comparisons
☐	☒	A full description of the statistical parameters including central tendency (e.g. means) or other basic estimates (e.g. regression coefficient) AND variation (e.g. standard deviation) or associated estimates of uncertainty (e.g. confidence intervals)
☐	☒	For null hypothesis testing, the test statistic (e.g. *F*, *t*, *r*) with confidence intervals, effect sizes, degrees of freedom and *P* value noted<br>*Give P values as exact values whenever suitable.*
☒	☐	For Bayesian analysis, information on the choice of priors and Markov chain Monte Carlo settings
☒	☐	For hierarchical and complex designs, identification of the appropriate level for tests and full reporting of outcomes
☒	☐	Estimates of effect sizes (e.g. Cohen's *d*, Pearson's *r*), indicating how they were calculated

Our web collection on statistics for biologists contains articles on many of the points above.

Software and code

Policy information about availability of computer code

Data collection	FACS Diva v5.0.3 and v8
Data analysis	geno2pheno[coreceptor] at https://coreceptor.geno2pheno.org/<br>geno2pheno[454] at https://454.geno2pheno.org/<br>FlowJo v7, v9, v10.5 and v10.7<br>GraphPad Prism v7<br>IUPM calculator at http://silicianolab.johnshopkins.edu/<br>HALO image analysis platform v3.2.1851.393

For manuscripts utilizing custom algorithms or software that are central to the research but not yet described in published literature, software must be made available to editors and reviewers. We strongly encourage code deposition in a community repository (e.g. GitHub). See the Nature Portfolio guidelines for submitting code & software for further information.

Data

Policy information about availability of data

All manuscripts must include a data availability statement. This statement should provide the following information, where applicable:

- Accession codes, unique identifiers, or web links for publicly available datasets
- A description of any restrictions on data availability
- For clinical datasets or third party data, please ensure that the statement adheres to our policy

Data Availability:
Figures 1, 2, and Extended Data Figures 1-5 contain raw data. HIV-1 sequence data is available via the NCBI GenBank® database (accession codes OP712709 - OP713600). The other data that support the findings of this study are available upon request via email to the corresponding authors B.-E.O.J. (bjoern-erikole.jensen@med.uni-duesseldorf.de) and J.S.z.W (j.schulze-zur-wiesch@uke.de) within the data protection constraints in the written informed consent signed by the study participants (i.e., pseudonymized data only), and will be made available within six weeks. The data are not publicly available as they contain information that could compromise the participants' privacy.

Human research participants

Policy information about studies involving human research participants and Sex and Gender in Research.

Reporting on sex and gender	n = 1 male individual who received CCR5 d32/d32 hematopoietic stem cells from a female individual.<br>n = 8 HIV-1 negative controls (37.5% female and 62.5% male sex based on self-reporting) and n = 1 HIV-1 positive control (male) for flow cytometric analyses.<br>n = 2 HIV-1 negative controls (both male) and n = 2 HIV-1 positive controls (one male, one female) for ISH analyses and immunohistochemistry.<br><br>Sex bias could not be considered in this study since this case report solely focuses on one single individual. However, the current literature on HIV cure in the context of CCR5Δ32/Δ32 HSCT does not hint at relevant sex-biased effects.
Population characteristics	n = 1 HIV-1 positive 53-year-old male individual after CCR5 d32/d32 HSCT.<br>n = 8 HIV-1 negative controls (37.5% female sex based on self-reporting; median age 25.5 years, range 21-28; 1 diagnosed with IBD, the others disclosed no comorbidities) and n = 1 HIV-1 positive control (69-year-old male; no disclosed comorbidities) for flow cytometric analyses.<br>n = 2 HIV-1 negative controls (gut biopsy of a 76-year-old male undergoing screening colonoscopy; lymph node of a 41-year-old female with adenoid cycstic carcinoma) and n = 2 HIV-1 positive controls (56-year-old male; 30-year-old male) for ISH analyses and immunohistochemistry.
Recruitment	Enrollment of the described subject at the University Medical Center Düsseldorf as IciStem #19. Enrollment of flow cytometry controls at the University Medical Center Hamburg-Eppendorf. Enrollment of ISH and IHC controls at Ghent University and OHSU Knight BioLibrary. All subjects were enrolled on a voluntary basis, were not compensated, and provided written informed consent complying with the Declaration of Helsinki principles.
Ethics oversight	Local ethics board of the Medical Faculty of the Heinrich Heine University Düsseldorf, Germany (Nr. 4261) for IciStem #19. Ärztekammer Hamburg, Germany (PV4780 and MC-316/14) for flow cytometry controls. Ethics committee of Ghent University Hospital, Belgium (BC-00812 and BC-11798) and Oregon Health & Science University Institutional Review Board, USA (IRB00004918) for ISH and IHC controls.

Note that full information on the approval of the study protocol must also be provided in the manuscript.

Field-specific reporting

Please select the one below that is the best fit for your research. If you are not sure, read the appropriate sections before making your selection.

☒ Life sciences ☐ Behavioural & social sciences ☐ Ecological, evolutionary & environmental sciences

For a reference copy of the document with all sections, see nature.com/documents/nr-reporting-summary-flat.pdf

Life sciences study design

All studies must disclose on these points even when the disclosure is negative.

Sample size	Sample size calculation was not applicable since this was a case study (n = 1). Sample size of controls (n = 8 HIV-1 negative controls and n = 1 HIV-1 positive control for flow cytometric analyses; n = 2 HIV-1 negative controls and n = 2 HIV-1 positive controls for ISH analyses and immunohistochemistry) was sufficient for comparison to the current data in the light of previous experiences (Eberhard et al. Sci. Transl. Med. 2020 and Deleage et al. JCI Insight 2016). Controls were intended to provide a reference to compare the data to.
Data exclusions	No data exclusions.

Replication

Biological replicates (i.e., samples at different time points of the patient) were measured in all experiments except ISH, IHC, and CCR5-PCR given the limited clinical material, tropism analysis given lack of viral sequences due to undetectable (pro-)viral loads, and mVOAs given ethical concerns and limited clinical material. Technical replicates (i.e., repeated measuring of the same sample) were measured as follows: for clinical viral load testing at each time point, two separate plasma samples were collected and measured independently from one another with similar results; for ddPCR, technical replicates were measured as indicated in Extended Data Table 2 with similar results (any divergent results are reported due to extremely rare abundance of HIV-1 DNA traces); for mVOAs, n = 2 and n = 5 mice were transplanted with the tested cells and generated similar results; ISH, IHC and flow cytometric experiments were not technically replicated given the limited clinical material; CCR5-PCR was done from PBMC ex vivo and 2 different T-cell expansions with similar results; most HIV-1-specific antibody immunoblots were measured once due to limited clinical material. The conclusiveness comes from coherent results due to periodic measurements.

Randomization

Randomization was not applicable since this was a case study on a single patient.

Blinding

Blinding was not applicable since this was a case study on a single patient.

Reporting for specific materials, systems and methods

We require information from authors about some types of materials, experimental systems and methods used in many studies. Here, indicate whether each material, system or method listed is relevant to your study. If you are not sure if a list item applies to your research, read the appropriate section before selecting a response.

Materials & experimental systems

n/a	Involved in the study
☐	☒ Antibodies
☐	☒ Eukaryotic cell lines
☒	☐ Palaeontology and archaeology
☐	☒ Animals and other organisms
☒	☐ Clinical data
☒	☐ Dual use research of concern

Methods

n/a	Involved in the study
☒	☐ ChIP-seq
☐	☒ Flow cytometry
☒	☐ MRI-based neuroimaging

Antibodies

Antibodies used

Immunophenotyping and MHC class I Tetramer staining:
CCR5 2D7 BUV737 1:100 BD Biosciences 565293
CD56 NCAM16.2 BUV737 1:1000 BD Biosciences 564448
CD45RA HI100 BUV737 1:800 BD Biosciences 564442
CD38 HB-7 BUV395 1:400 BD Biosciences 563811
CD8a RPA-T8 BV785 1:1000 BioLegend 301046
Tim-3 F38-2E2 BV785 1:100 BioLegend 345031
HLA-DR L243 BV711 1:800 BioLegend 307644
CD8a RPA-T8 BV711 1:200 BioLegend 301043
CD45RA HI100 BV650 1:800 BioLegend 304136
CD16 3G8 BV650 1:400 BioLegend 302042
PD-1 EH12.2H7 BV650 1:80 BioLegend 329949
CXCR4 12G5 BV605 1:200 BioLegend 306522
TCR Vα7.2 3C10 BV605 1:400 BioLegend 351719
TIGIT A15153G BV605 1:130 BioLegend 372712
CD3 UCHT1 BV510 1:200 BioLegend 300448
CCR7 G043H7 BV421 1:400 BioLegend 353207
NKG2D 1D11 BV421 1:100 BioLegend 320822
CD39 A1 BV421 1:1000 BioLegend 328214
CD4 SK3 PerCP-Cy5.5 1:2000 BioLegend 344607
CD127 A019D5 PerCP-Cy5.5 1:100 BioLegend 351322
CD27 M-T271 FITC 1:200 BD Biosciences 555440
TCR Vδ2 B6 FITC 1:200 BioLegend 331406
CD25 M-A251 PE 1:400 BioLegend 356104
TCRγδ 11F2 PE 1:50 BD Biosciences 333141
PD-1 EH12.2H7 PE-Dazzle594 1:400 BioLegend 329939
CD161 HP-3G10 PE-Dazzle594 1:200 BioLegend 339940
CD38 HB-7 PE-Cy7 1:800 BioLegend 356608
CCR7 G043H7 PE-Cy7 1:130 BioLegend 353226
CD3 UCHT1 AF700 1:100 BioLegend 300424
HLA-DR L243 AF700 1:130 BioLegend 307625
CD127 A019D5 AF647 1:400 BioLegend 351317
CD14 63D3 APC-Cy7 1:800 BioLegend 367108
CD19 HIB19 APC-Cy7 1:800 BioLegend 302218

ICS:

	CD3 UCHT1 AF700 1:15 BD Biosciences 557943<br>CD4 RPA-T4 APC 1:15 BD Biosciences 555349<br>CD8a RPA-T8 APC-Cy7 1:15 BD Biosciences 557760<br>CD107a H4A3 V450 1:10 BD Biosciences 561345<br>IFN-γ B27 PE-Cy7 1:14 BD Biosciences 557643<br>IL-2 5344.111 FITC 1:5 BD Biosciences 340448<br>TNF-α Mab11 PE-CF594 1:10 BD Biosciences 562784<br><br>Cell Sorting for ddPCR:<br>CD3 UCHT1 APC 1:20 BD Biosciences 555335<br>CD3 OKT3 eFluor450 1:50 eBioscience 48-0037-42<br>CD4 RPA-T4 APC-eFluor780 1:50 eBioscience 47-0049-42<br>CD8 SK1 APC-H7 1:40 BD Biosciences 560179<br>CD8 SK1 PerCP 1:20 BD Biosciences 345774<br>CD14 HCD14 PerCP-Cy5.5 1:50 BioLegend 325622<br>CD27 O343 APC 1:50 eBioscience 17-0279-42<br>CD45 HI30 FITC 1:20 BD Biosciences 555482<br>CD45RA L48 FITC 1:10 BD Biosciences 335039<br>CD45RO UCHL1 PE-Cy7 1:50 BD Biosciences 337168<br>CD95 DX2 BV711 1:25 BD Biosciences 555674<br>CXCR5 J252D4 PE-Cy7 1:80 BioLegend 356924<br>CCR7 3D12 PE 1:50 BD Biosciences 552176<br>PD-1 EH12.1 BV421 1:20 BD Biosciences 562516<br><br>IHC:<br>CD4 EPR6855 1:200 Abcam ab133616<br>CD68 KP1 1:200 Biocare CM033C<br>CD163 10D6 1:600 ThermoFisher MA5-11458<br>MPO polyclonal 1:5000 Dako A0398<br>Mx-1 M143 1:1000 EMD MABF938
Validation	All antibodies had a validated technical data sheet as per the manufacturer's website. All antibodies were titrated for their optimal dilution.

Eukaryotic cell lines

Policy information about cell lines and Sex and Gender in Research

Cell line source(s)	TZM-bl, U373-MAGI-CCR5E and U373-MAGI-CXCR4CEM were obtained from the NIH HIV reagents program.
Authentication	None of the cell lines has been authercticated.
Mycoplasma contamination	Cell lines tested negative for mycoplasma.
Commonly misidentified lines<br>(See ICLAC register)	None of the cell lines is listed in the ICLAC database.

Animals and other research organisms

Policy information about studies involving animals; ARRIVE guidelines recommended for reporting animal research, and Sex and Gender in Research

Laboratory animals	6-week-old Balb/c Rag2-/-γc-/- mice, mixed sex; 7-week-old NOD.Cg-Prkdcscid Il2rgtm1Wjl/SzJ (NSG) mice, mixed sex.
Wild animals	No involvement of wild animals in the study.
Reporting on sex	Sex has not been considered in the study design for murine assays, since the immunodeficient mice were used as a "tool" for viral rescue. Therefore, sex of the animals has not been willingly assigned to patient or control assays. However, both male and female mice were used in the assays.
Field-collected samples	No involvement of field-collected samples in the study.
Ethics oversight	Ärztekammer Hamburg (OB-050/07 and WF-010/2011) and Freie und Hansestadt Hamburg, Behörde für Gesundheit und Verbraucherschutz (Nr.: 63/09 and 23/11) for Balb/c Rag2-/-γc-/- mice. Animal Experimentation Ethics Committee of the University Hospital Germans Trias i Pujol (registered as B9900005) for NOD.Cg-Prkdcscid Il2rgtm1Wjl/SzJ (NSG) mice.

Note that full information on the approval of the study protocol must also be provided in the manuscript.

Flow Cytometry

Plots

Confirm that:

☒ The axis labels state the marker and fluorochrome used (e.g. CD4-FITC).

☒ The axis scales are clearly visible. Include numbers along axes only for bottom left plot of group (a 'group' is an analysis of identical markers).

☒ All plots are contour plots with outliers or pseudocolor plots.

☒ A numerical value for number of cells or percentage (with statistics) is provided.

Methodology

Sample preparation	PBMC were isolated by density gradient centrifugation from whole blood samples and stored at stored at −196 °C. Thawed PBMC were washed in PBS, and surface stained for 30 min with saturating concentrations of different combinations of antibodies in the presence of fixable live/dead stain. For ICS, cells were then fixed and permeabilized for detection of intracellular antigens.
Instrument	LSR Fortessa and LSRII (BD Biosciences)
Software	FlowJo v10.5 and v10.7
Cell population abundance	Sort of peripheral blood cells:<br>M37 410,000 Tn; 840,000 Tcm; 360,000 Ttm; 540,000 Tem<br>M80 230,000 CD4+ T cells; 550,000 Tn; 420,000 Tcm; 310,000 Ttm; 330,000 Tem<br>M94 520,000 CD4+ T cells<br>M102 340,000 CD4+ T cells<br>M111 380,000 CD4+ T cells<br>M114 2,100,000 CD4+ T cells<br>Purity of CD3+ CD4+ cells >95% as determined by flow cytometric analysis.<br><br>Sort of tissue cells:<br>M37 ileum 25,000 LPL; rectum 54,000 LPL<br>M51 lymph node 1,500 Tfh cells<br>M77 duodenum 215,000 LPL; ileum 105,000 LPL; rectum 83,000 LPL<br>No purity assessments performed.<br><br>See also Ext. Data Table 2 for cell population abundance (= cells used in the PCR assays).
Gating strategy	Cells were distinguished from debris based on FCS-A and SSC-A. Doublets were excluded based on FSC-A and FSC-H. Live cells were distinguished from dead cells by negative selection using LIVE/DEAD Fixable Aqua Dead Cell Stain kit (Thermo Fisher Scientific) or Zombie NIR fixable viability dye (BioLegend).<br>T cells and T-cell subsets were defined based on expression of CD3, CD4, CD8, CCR7, CD45RA, CD45RO, CD27, TCRγδ, CD161 and TCR Vα7.2. NK cells and NK cell subsets were defined based on expression of CD16 and CD56. See also supplementary data for gating strategy.

☒ Tick this box to confirm that a figure exemplifying the gating strategy is provided in the Supplementary Information.
