# Supplementary material for: In-depth virological and immunological characterization of HIV-1 cure after CCR5Δ32/Δ32 allogeneic hematopoietic stem cell transplantation
Source: Nat Med. 2023 Feb 20;29(3):583–7. doi: 10.1038/s41591-023-02213-x (PMC10033413; doi:10.1038/s41591-023-02213-x)
Supplement: Supplementary file 3 — Full immunoblot images. [file 41591_2023_2213_MOESM3_ESM.pdf]

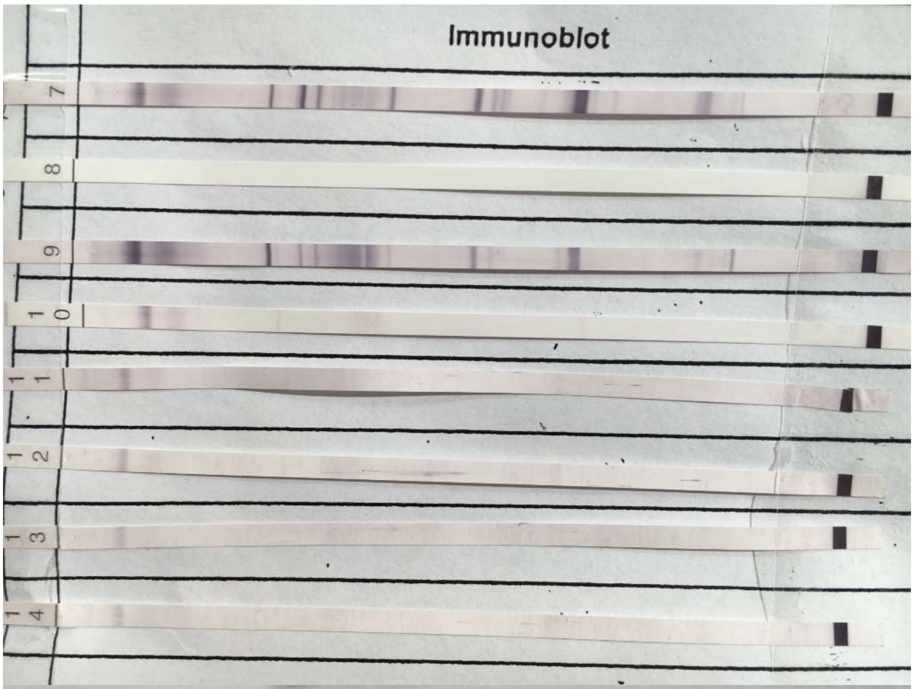


positive ctrl
negative ctrl
02/2013 (M+0)
06/2014 (M+16)
02/2015 (M+24)
07/2015 (M+29)
12/2015 (M+34)
01/2016 (M+35)

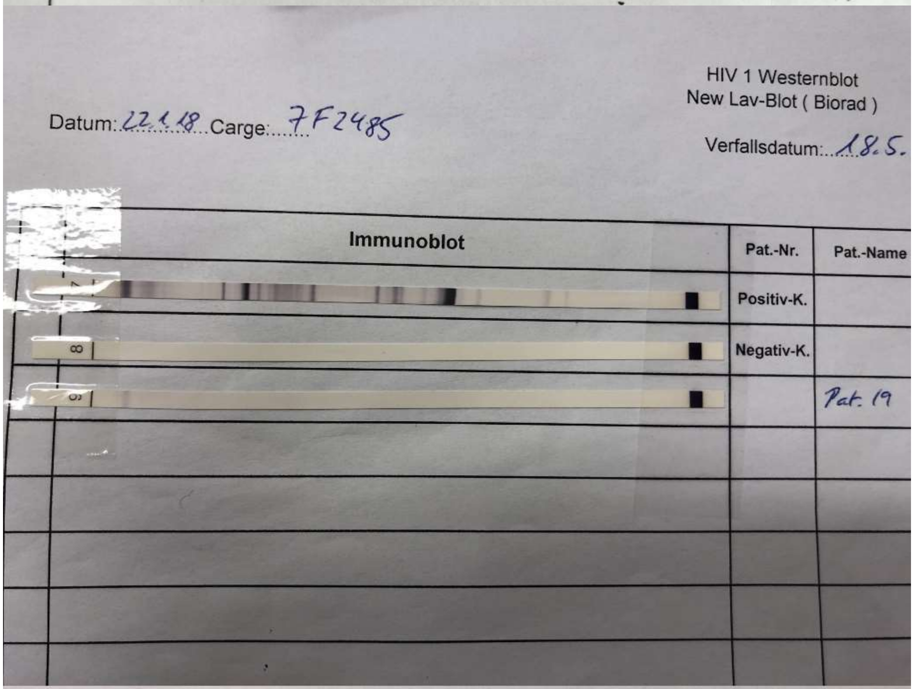


positive ctrl
negative ctrl
01/2018 (M+59)

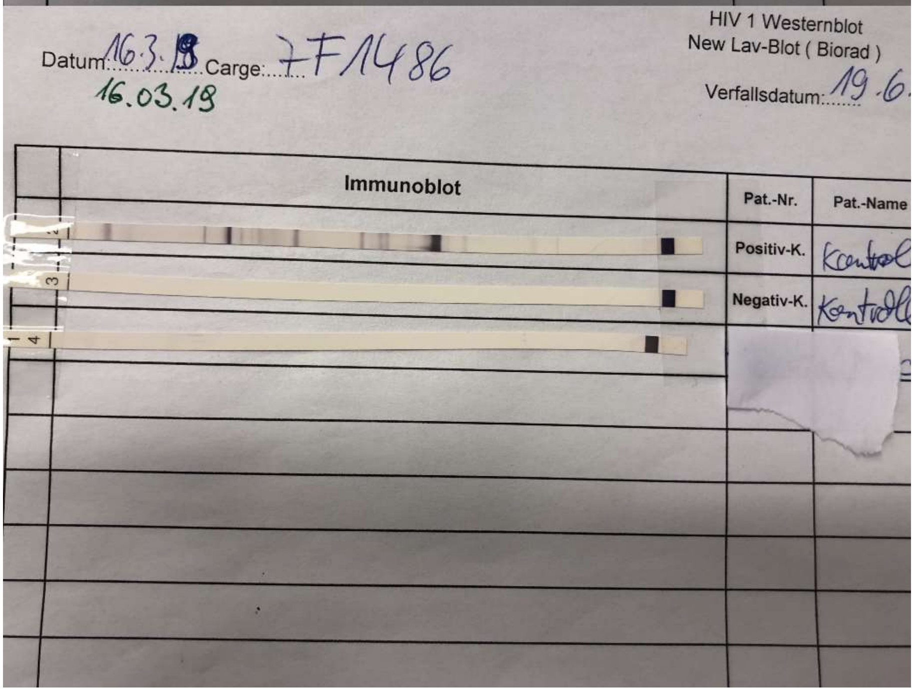


positive ctrl
negative ctrl
03/2019 (M+73)

**Source Data for Fig. 2d:**
**Full immunoblot images.**
