# Supplementary material for: In-depth virological and immunological characterization of HIV-1 cure after CCR5Δ32/Δ32 allogeneic hematopoietic stem cell transplantation
Source: Nat Med. 2023 Feb 20;29(3):583–7. doi: 10.1038/s41591-023-02213-x (PMC10033413; doi:10.1038/s41591-023-02213-x)
Supplement: Supplementary file 4 — Full gel electrophoresis. [file 41591_2023_2213_MOESM4_ESM.pdf]

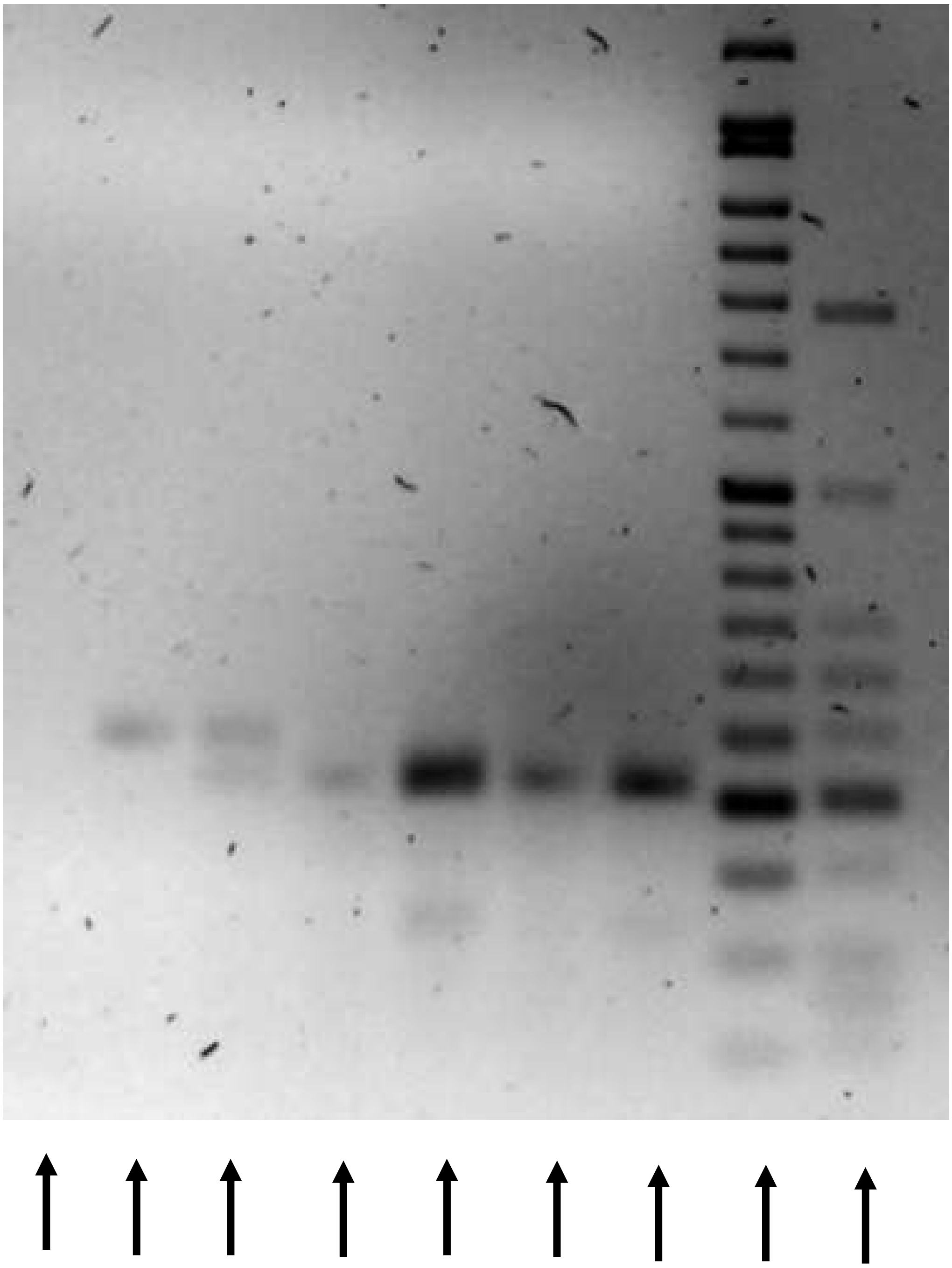

1 2 3 4 5 6 7 8 9

1 – water control
2 – CCR5wt/wt donor
3 – CCR5Δ32/wt donor
4 – CCR5Δ32/Δ32 donor
5 – IciStem #19 PBMC M+59
6 – IciStem #19 RT-YV9 CTL line
7 – IciStem #19 Nef-FL9 CTL line
8 – 50bp DNA ladder
9 – low molecular weight DNA ladder

Source Data for Extended Data Fig. 1b:
Full gelelectrophoresis
